# OTUD1 Prevents Immunosuppression via Promoting Lysosomal‐Associated Degradation of PD‐L1

**DOI:** 10.1002/mco2.71144

**Published:** 2026-11-08

**Authors:** Yang Hao, Hengzong Zhou, Yanxin Liu, Minjun Jin, Qiang Feng, Tiantian Li, Qirong Li, Yahui Zhen, Tong Sun, Chenhao Li, Dongxu Wang

**Affiliations:** ^1^ Laboratory Animal Center, College of Animal Science Jilin University Changchun China; ^2^ Department of Gastric and Colorectal Surgery, General Surgery Center The First Hospital of Jilin University Changchun China; ^3^ Department of Breast Surgery China‐Japan Union Hospital of Jilin University Changchun China; ^4^ Department of Nephrology, Lequn Branch The First Hospital of Jilin University Changchun China

**Keywords:** autophagy, kidney renal clear cell carcinoma, lysosome, OTUD1, PD‐L1

## Abstract

Upregulation of PD‐L1 on tumor cells is a pivotal aspect of immune evasion, leading to treatment resistance, tumor recurrence, and metastasis. Understanding the mechanisms that control PD‐L1 expression in cancer cells is therefore of paramount importance. In this study, we show that deubiquitinase OTUD1 is downregulated in kidney renal clear cell carcinoma (KIRC), and its low expression is linked to a poor prognosis in patients. Analysis of a public single‐cell sequencing dataset revealed that OTUD1 is enriched in specific immune cell populations, indicating its potential for immune modulation. Overexpression of OTUD1 significantly hindered KIRC progression and was associated with decreased PD‐L1 expression in both KIRC patients and mouse cancer models. Further investigation uncovered that OTUD1 downregulates PD‐L1 abundance and enhances tumor cell sensitivity to cytotoxicity mediated by Jurkat T cells. Mechanistically, OTUD1 interacts with and deubiquitinates the protein kinase AKT, thereby promoting the autophagosome‐lysosomal degradation of PD‐L1 via the AKT‐mTOR‐TFEB axis. Moreover, OTUD1 overexpression induced autophagy and enhanced the release or exposure of DAMPs, thereby promoting T cell infiltration into the tumor microenvironment in mice. Our study unveils a novel mechanism by which OTUD1 regulates PD‐L1 degradation, highlighting its potential as a therapeutic target in KIRC cancer immunology.

## Graphical abstract

OTUD1 interacts with AKT and decreases AKT‐mTOR pathway activity, facilitating the translocation of TFEB to the nucleus, thereby triggering autophagy‐dependent degradation of PD‐L1 in lysosomes.

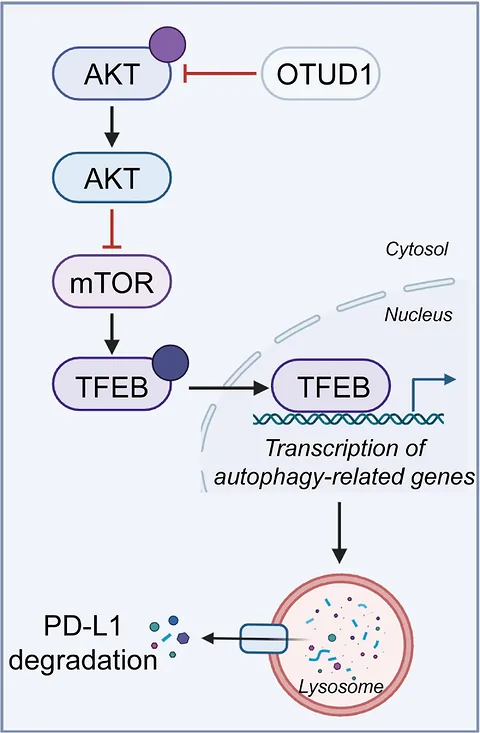

## 1 Introduction

Kidney renal clear cell carcinoma (KIRC) is the most common histological subtype of renal cell carcinoma, accounting for approximately 75%–80% of all malignant kidney tumors. It is characterized by a high potential for metastasis, and patients with advanced disease have a poor clinical prognosis [1]. Although significant progress has been made in surgical and targeted therapies, a substantial proportion of patients ultimately experience recurrence or distant metastasis, limiting long‐term survival rates [1]. Moreover, KIRC frequently shows high immunogenicity and heavy immune cell infiltration in the tumor, coexisting with adaptive immune resistance, particularly via PD‐1/PD‐L1 inhibitory pathways [2, 3, 4]. Regimens targeting PD‐1/PD‐L1 have been approved by the U.S. Food and Drug Administration (FDA) for the treatment of advanced kidney cancer, with combination strategies utilizing anticytotoxic T‐lymphocyte antigen 4 (CTLA‐4) or vascular endothelial growth factor (VEGF) tyrosine kinase receptor inhibitors rapidly becoming first‐line therapies [5]. Studies involving metastatic renal cell carcinoma (RCC/KIRC) patients revealed that tumor PD‐L1 expression can be recognized as a predictive biomarker for the efficacy of Nivolumab (anti‐PD‐1) treatment [6]. Although immunohistochemical detection of PD‐L1 has been established as a selection criterion for patients receiving anti‐PD‐1/PD‐L1 therapy, not all PD‐L1‐positive patients respond well [7], underscoring the complexity of its regulatory network. This counteracting antibody efficacy may be due to the compensatory upregulation of PD‐L1 and its subcellular relocalization from the endoplasmic reticulum, Golgi apparatus, and endosomes to the tumor cell membrane [8]. Thus, elucidating the molecular mechanisms governing PD‐L1 regulation is crucial for understanding KIRC immune evasion.

High PD‐L1 levels in tumors are tightly regulated by genomic alterations, epigenetic mechanisms, posttranslational modifications, and dysregulated oncogenic and inflammatory signaling pathways [9, 10]. Deubiquitylases (DUBs) regulate ubiquitin‐dependent pathways by catalyzing the hydrolysis of ester, peptide, or isopeptide bonds between ubiquitin and substrate proteins. In eukaryotes, approximately 100 DUBs have been identified and categorized into seven major families based on domain architecture and sequence conservation [11]. They play a role in nearly all cellular biological processes, including protein homeostasis regulation, signal transduction, cell cycle control, and gene expression regulation. In recent years, the role of DUBs in tumorigenesis and tumor immunity has garnered significant attention [12]. Specific DUBs, including ubiquitin‐specific protease (USP)22, CSN5, OTUB1, and USP5, can promote immune escape by binding to and stabilizing tumor PD‐L1 [13]. OTU deubiquitinase 1 (OTUD1), a cytosolic DUB that belongs to the ovarian tumor domain (OTU) family, contains multiple highly conserved domains [14]. Dysregulation of OTUD1 is linked to poor prognosis and increased tumor burden in multiple myeloma (MM), non–small cell lung cancer (NSCLC), and breast cancer. It has been demonstrated that OTUD1 facilitates ferroptosis by deubiquitinating and stabilizing iron‐responsive element‐binding protein 2 (IREB2), thereby promoting infiltration of cytotoxic T lymphocytes (CTLs) in the tumor microenvironment (TME) and reducing PD‐1 surface expression [15]. OTUD1 was reported to regulate immune evasion in colorectal cancer by targeting fibrinogen‐like protein 1 (FGL‐1), a novel immune checkpoint molecule [16]. Previous studies have demonstrated that OTUD1 participates in regulating tumor immunity through STAT3‐dependent regulation of PD‐L1 transcription [17]. However, whether OTUD1 regulates PD‐L1 protein stability through posttranslational mechanisms remains unclear.

Emerging evidence highlights lysosome‐dependent autophagy as a critical regulator of PD‐L1 protein stability and anticancer immunity. For instance, huntingtin‐interacting protein 1‐related (HIP1R) and microphthalmia‐associated transcription factor (MITF) directly interact with PD‐L1, promoting PD‐L1 lysosomal degradation to enhance T‐cell‐mediated cytotoxicity [18, 19], whereas Chemokine‐like factor (CKLF)‐like MARVEL transmembrane structural domain‐containing protein 6 (CMTM6) inhibits PD‐L1 degradation via the lysosomal process [20]. Furthermore, naturally derived Tubeimoside‐1 (TBM‐1) induces transcriptional factor EB (TFEB)‐dependent PD‐L1 degradation via mammalian target of rapamycin protein complex 1 (mTORC1) inhibition, thereby alleviating the immunosuppressive microenvironment and synergizing with CTLA‐4 inhibitors in Lewis lung carcinoma and melanoma [21]. Despite the existing knowledge, the role of autophagy in regulating PD‐L1 in cancer cells warrants further investigation.

In the present study, we first characterized the expression pattern and clinical relevance of OTUD1 in KIRC and determined its effects on tumor‐cell proliferation, cell‐cycle progression, migration, invasion, and tumor growth. We subsequently investigated whether OTUD1 deficiency is associated with an immune‐evasive tumor microenvironment and whether OTUD1 directly regulates PD‐L1 abundance in KIRC cells. Mechanistically, we examined the role of the AKT‐mTOR‐TFEB axis and autophagy‐lysosomal pathway in OTUD1‐mediated PD‐L1 degradation. Our findings identify OTUD1 as a regulatory molecule involved in tumor suppression and immune regulation in KIRC and provide new insights into the posttranslational regulation of PD‐L1.

## 2 Results

### 2.1 OTUD1 Is Downregulated in KIRC and Associated With Unfavorable Clinical Outcome

To investigate the expression and clinical relevance of OTUD1 in renal cell carcinoma, we analyzed transcriptomic data from the TCGA and GTEx databases using the TIMER online website (http://timer.cistrome.org/) and the XIANTAO tool (https://www.xiantao.love/). OTUD1 expression was significantly altered in 15 cancer types, with lower levels frequently observed in tumors compared with the corresponding normal tissues (Figure 1A,B). Notably, we found that OTUD1 was significantly downregulated in KIRC tumors relative to their unpaired or paired precancerous samples (Figure 1C,D). The violin plot revealed progressively decreasing OTUD1 levels with advancing pathological stage, suggesting a correlation between OTUD1 expression and disease progression (Figure 1E). Moreover, metastatic KIRC cell lines (e.g., Caki‐1 and SLR 21, etc.) exhibited lower OTUD1 expression than primary tumor‐derived cells (e.g., 786‐O, A‐498, and Caki‐2, etc.) (Figure 1F; Figure S1A). The underlying causes of these basal expression differences may include intrinsic genetic mutations or epigenetic alterations.

**FIGURE 1:**
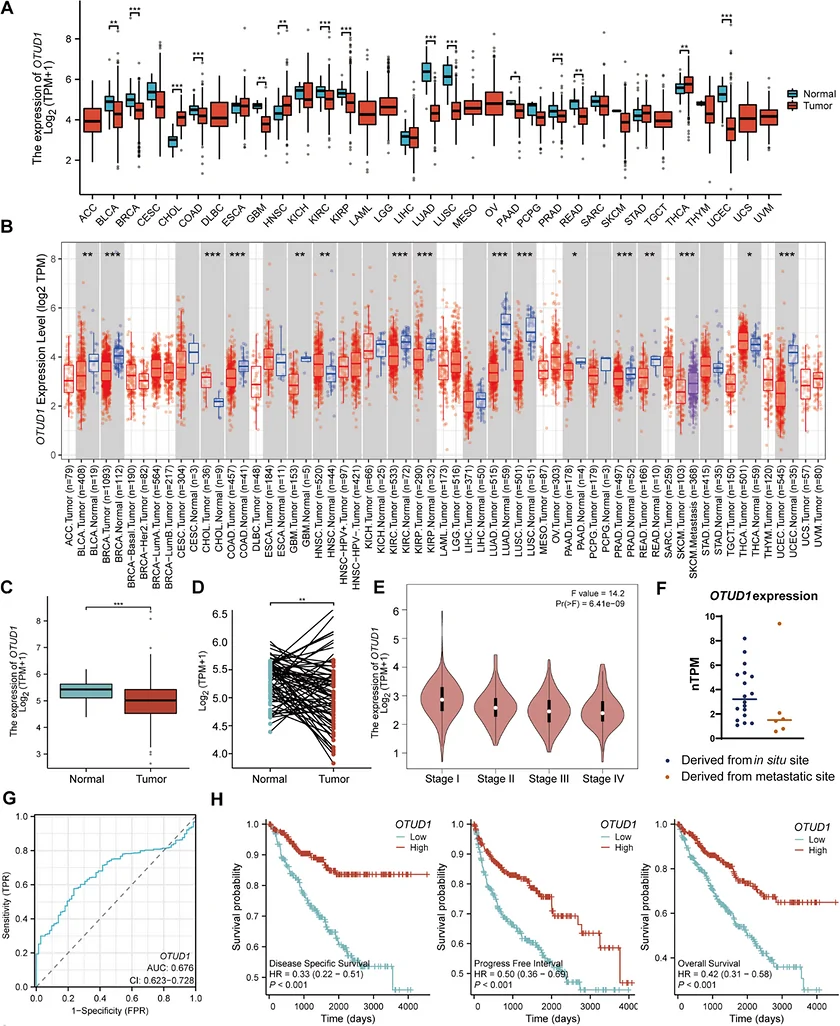
The clinical significance of OTUD1 in KIRC patients. (A, B) Expression levels of *OTUD1* in different cancer types from the TIMER and XIANTAO databases. (C) Expression levels of OTUD1 in tumor tissues and paraneoplastic tissues of KIRC patients. (D) Expression levels of OTUD1 in KIRC tumor tissues and adjacent carcinoma tissues of paired samples in the TCGA dataset. (E) Expression of *OTUD1* in patients with different tumor stages of KIRC (GEPIA). (F) Statistical analysis of RNA expression (normalized transcripts per million [nTPM]) of *OTUD1* in cell lines derived from metastatic sites () and in situ sites. (G) The ROC (receiver‐operating characteristic) analysis of *OTUD1* as a diagnostic marker in KIRC patients in TCGA. AUC, area under the curve. (H) Relationship of *OTUD1* expression levels with disease‐specific survival (DSS), progression‐free interval (PFI), and overall survival (OS) in the TCGA‐KIRC cohort. The statistical difference between the two groups was compared using the Wilcox test; differences were considered significant at *p* < 0.05 (*p < 0.05, **p < 0.01, ***p < 0.001).

Furthermore, we evaluated the clinical prognostic value of OTUD1 in KIRC. The diagnostic/prognostic value of OTUD1 was analyzed, and receiver operating characteristic (ROC) curve analysis [22] yielded an area under the curve (AUC) of 0.676, indicating that OTUD1 expression had a moderate predictive performance for distinguishing KIRC patients from healthy individuals (Figure 1G). Although multivariate Cox regression analysis did not show a significant correlation between OTUD1 expression and OS in KIRC patients, there was significance in univariate Cox regression analysis. (Figure S1B–C). This can be explained by our previous data that OTUD1 expression progressively and significantly decreases with advancing clinical stage and higher histological grade (Figure 1E). In addition, Kaplan–Meier (KM) survival analysis demonstrated that low OTUD1 expression was associated with significantly worse overall survival (OS), progression‐free interval (PFI), and disease‐specific survival (DSS) compared with those with high expression (Figure 1H). Overall, these results indicate that OTUD1 is frequently downregulated in KIRC and potentially correlates with advanced disease stage, poor prognosis, and antitumor immunity in KIRC patients.

### 2.2 OTUD1 Overexpression Suppresses KIRC Malignant Progression

To investigate the biological function of OTUD1 in KIRC progression, OTUD1 was overexpressed or silenced in KIRC cell lines. Overexpression of OTUD1 significantly suppressed the viability of 786‐O cells (Figure 2A), whereas OTUD1 deletion promoted tumor cell growth (Figure S2A). Cell cycle analysis following OTUD1 overexpression revealed an increased proportion of cells in the G0/G1 phase and a reduced number in the S phase, indicating that OTUD1 suppresses KIRC growth through inhibition of cell‐cycle progression (Figure 2B,C). Conversely, siRNA‐mediated OTUD1 knockdown decreased the G0/G1 population (Figure S2B,C). No significant difference in cell apoptosis was found between OTUD1‐overexpressing and control cells (Figure S2D). We next examined the effect of OTUD1 on metastatic behaviors and found that OTUD1 overexpression markedly impaired cell invasion, as evidenced by a decrease in the number of cells penetrating the Transwell membrane (Figure 2D,E). Similarly, the control group of 786‐O cells exhibited strong, aggressive motility, whereas overexpression of OTUD1 markedly reduced migratory capacity (Figure 2F; Figure S2E–G). We next examined the expression of major epithelial‐to‐mesenchymal (EMT)‐related proteins. OTUD1 upregulation downregulated the expression levels of mesenchymal markers, N‐Cadherin and Vimentin, while inducing the expression of the epithelial marker E‐Cadherin (Figure S3A,B). In contrast, downregulation of OTUD1 by specific siRNA‐mediated depletion showed an opposite trend in these EMT markers (Figure S3C, D).

**FIGURE 2:**
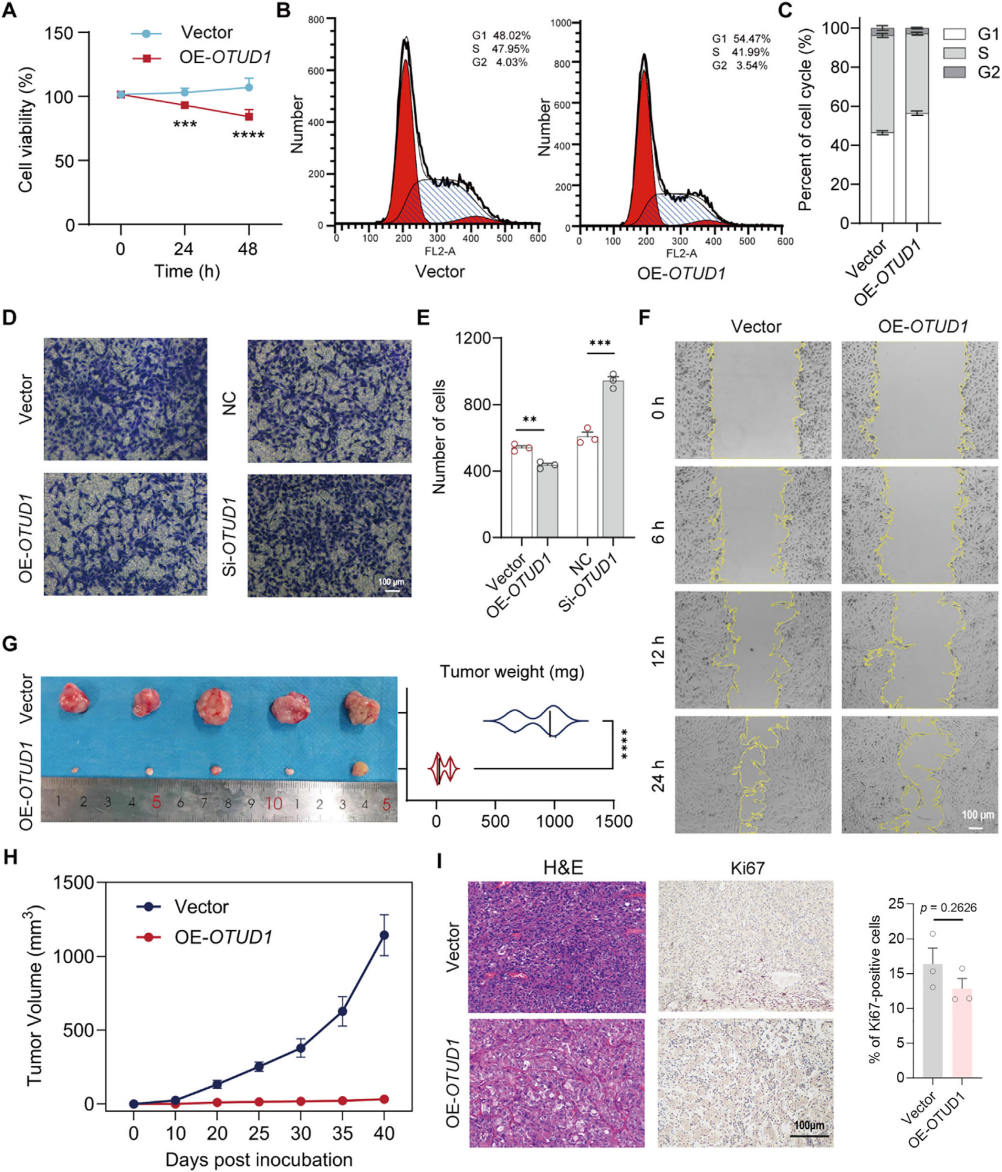
OTUD1 overexpression inhibits cancer progression in KIRC. Effects of overexpression of *OTUD1* on 786‐O cell viability, as measured by cell counting kit‐8 (CCK‐8) assays. (B, C) Representative flow cytometry analysis and quantitative analysis for cell cycle distribution of 786‐O cells. (D, E) The Transwell invasion assay and quantitative analysis revealed the effects of overexpressing or knocking down OTUD1 on cell migration. Representative images from two independent experiments are shown. Scale bars = 100 µm. (F) Effects of overexpression of OTUD1 on the migration ability of 786‐O cells by wound healing images and quantitative analysis. Representative images from two independent experiments are shown. Scale bars = 100 µm. (G) Stable OTUD1‐overexpressing 786‐O cells or 786‐O cells transfected with a negative vector as a control were injected into the right flank of nude mice. Tumor growth and mouse weight were monitored, followed by the collection of tumor nodules and recording of tumor weights at 40 days postinoculation. Representative gross images of tumors from each group and their final tumor weight are given. (H) Relative tumor growth curve of 786‐O cells with or without OTUD1 overexpression. (I) Immunohistochemical analysis for actively dividing cells by antigen Kiel 67 (Ki67) expression and Hematoxylin–eosin (H&E) staining of tumors in different groups of mouse models (100× magnification, Scale bar = 100 µm). Representative images from two independent experiments are shown. Significance was assessed using a two‐way ANOVA followed by Dunnett's (A, E) and Tukey's (H) multiple comparisons tests, and a two‐tailed unpaired Student's *t*‐test (G). Data are representative of at least three (B, D, F, I) independent experiments with similar results.

To confirm the antitumor function of OTUD1 in vivo, we employed a xenograft model in immunodeficient mice. The mice inoculated with OTUD1‐overexpressing tumors exhibited delayed growth onset and significantly smaller volumes at 40 days postinoculation compared with the control group (Figure 2G,H). Tumor weight was reduced by 94% (Figure 2G). Furthermore, OTUD1 overexpression promoted tumor cell death and suppressed tumor cell proliferation in vivo (Figure 2I). In summary, these results demonstrate that OTUD1 exerts tumor‐suppressive effects in KIRC by inhibiting proliferation, migration, invasion, and EMT in vitro, and attenuating tumor growth in vivo.

### 2.3 OTUD1 Expression Correlates With the Regulation of TME and the Posttranslational Regulation of PD‐L1

Increasing evidence indicates that immune escape plays a critical role in shaping cancer progression and therapeutic responses. Therefore, we analyzed the involvement of OTUD1 in the regulation of TME. Notably, OTUD1‐low tumors exhibited profound alterations in the overall TME, evidenced by dramatic shifts in the immune score (*p* < 0.0001) and microenvironment score (Figure 3A). Further detailed analysis revealed that OTUD1 status significantly associated with the infiltration abundance of major immune cell subsets. For instance, robust alterations were observed in the infiltration of CD8^+^ T cells, M2 macrophages, and regulatory T cells (Tregs) (Figure 3B). OTUD1 expression was also associated with immune subtypes linked to immunotherapy resistance (wound healing, transforming growth factor‐β (TGF‐β) dominant) and metastasis (lymphocyte‐depleted, immunologically quiet) (Figure 3C). Given that checkpoint is the key modulator of TME and immune escape in KIRC, we investigated the relationship between OTUD1 and immune checkpoint expression. KIRC patients with low OTUD1 expression displayed increased gene expression of eight key immune checkpoint molecules (Figure 3D).

**FIGURE 3:**
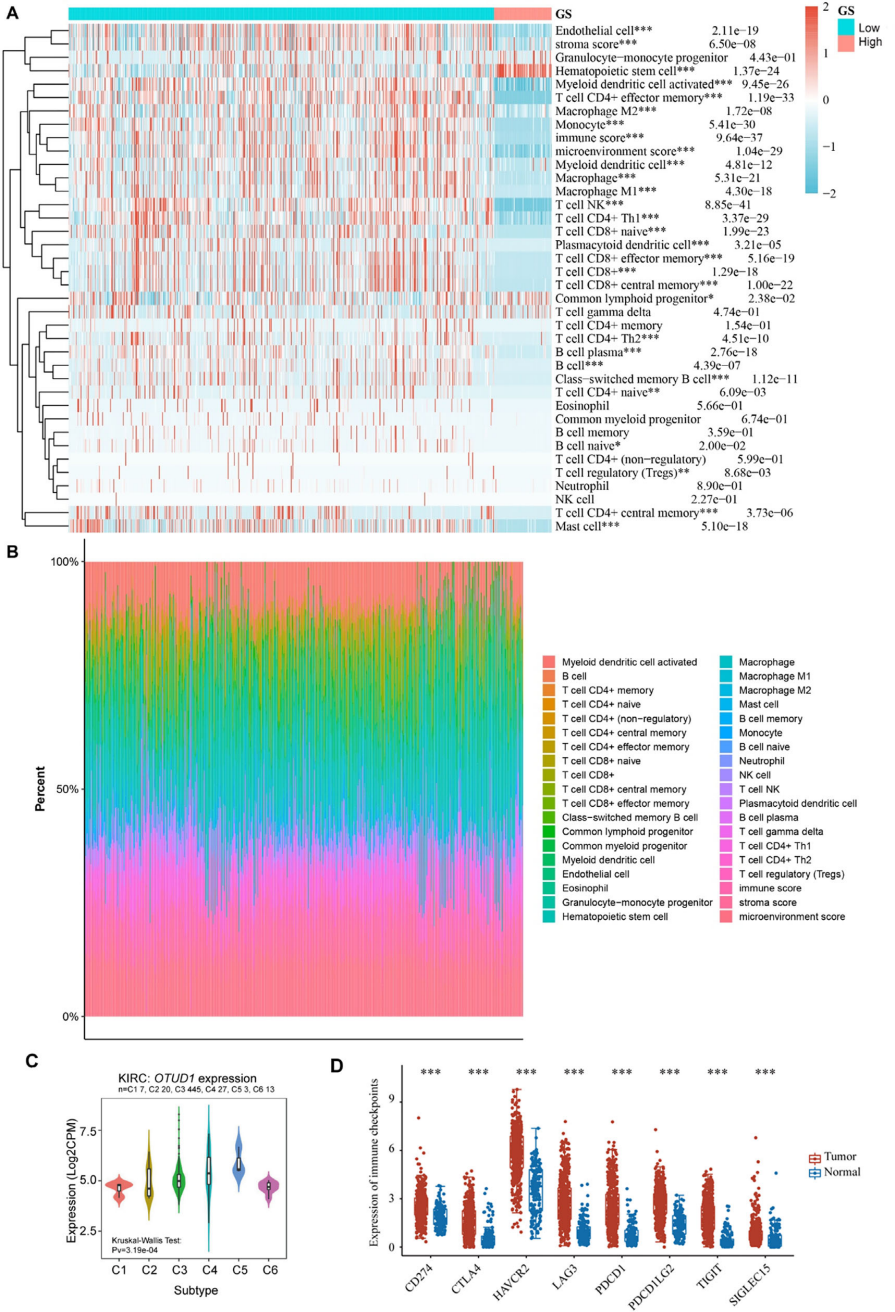
OTUD1 expression correlates with TME regulation and PD‐L1 expression in KIRC. (A) Immunoscore heatmap of immune cell scores with high and low OTUD1 expression in KIRC tumors and (B) percentage abundance of infiltrating immune cells for each sample. (C) The correlation of *OTUD1* expression and the immune subtypes of KIRC patients from the TISIDB database, including C1 (wound healing), C2 (IFN‐g dominant), C3 (inflammatory), C4 (lymphocyte depleted), C5 (immunologically quiet), and C6 (TGF‐β dominant). (D) The correlation of subgroups with high and low OTUD1 expression and immune checkpoint gene expression. The statistical difference between the two groups was compared using the Wilcox test. *p < 0.05, **p < 0.01, ***p < 0.001, ns *p* > 0.05.

Next, we sought to determine whether a correlation exists between OTUD1 and PD‐L1 in KIRC tissues. Consistent with bioinformatic analysis, western blot analysis of patient tissues revealed that low OTUD1 expression in KIRC tumors was associated with high PD‐L1 levels (Figure 4A). The immunofluorescence staining in Renca syngeneic mouse tumor models also showed that OTUD1 overexpression correlated with reduced PD‐L1 expression and increased intratumoral infiltration of CD3^+^, CD4^+^, and CD8^+^ T cells (Figure 4B; Figure S4). To clarify whether this relationship is correlative or regulatory, we examined PD‐L1 expression after overexpressing or downregulating OTUD1 in KIRC cell lines. The protein levels of PD‐L1 were significantly decreased following OTUD1 overexpression, whereas OTUD1 knockdown increased PD‐L1 abundance in both 786‐O and Renca cells (Figure 4C). Since membrane‐localized PD‐L1 is functionally critical for immunosuppression [23], we evaluated whether OTUD1 influences its surface expression. Flow cytometry analysis results confirmed that overexpression reduced, while its knockdown enhanced, membrane PD‐L1 levels in 786‐O cells (Figure 4D) and Renca cells (Figure 4E). Furthermore, under stimulation with natural killer (NK) cell supernatants containing interferon (IFN)‐γ, known inducers of PD‐L1, OTUD1 overexpression still reduced membrane PD‐L1 expression but without a statistical difference in 786‐O cells and Renca cells. In contrast, OTUD1 knockdown significantly increased membrane PD‐L1 levels in both cell lines, indicating that OTUD1 can also downregulate PD‐L1 under inflammatory conditions (Figure 4F–I). Together, these findings demonstrate that OTUD1 negatively regulates PD‐L1 protein abundance in KIRC, both under basal and cytokine‐induced conditions.

**FIGURE 4:**
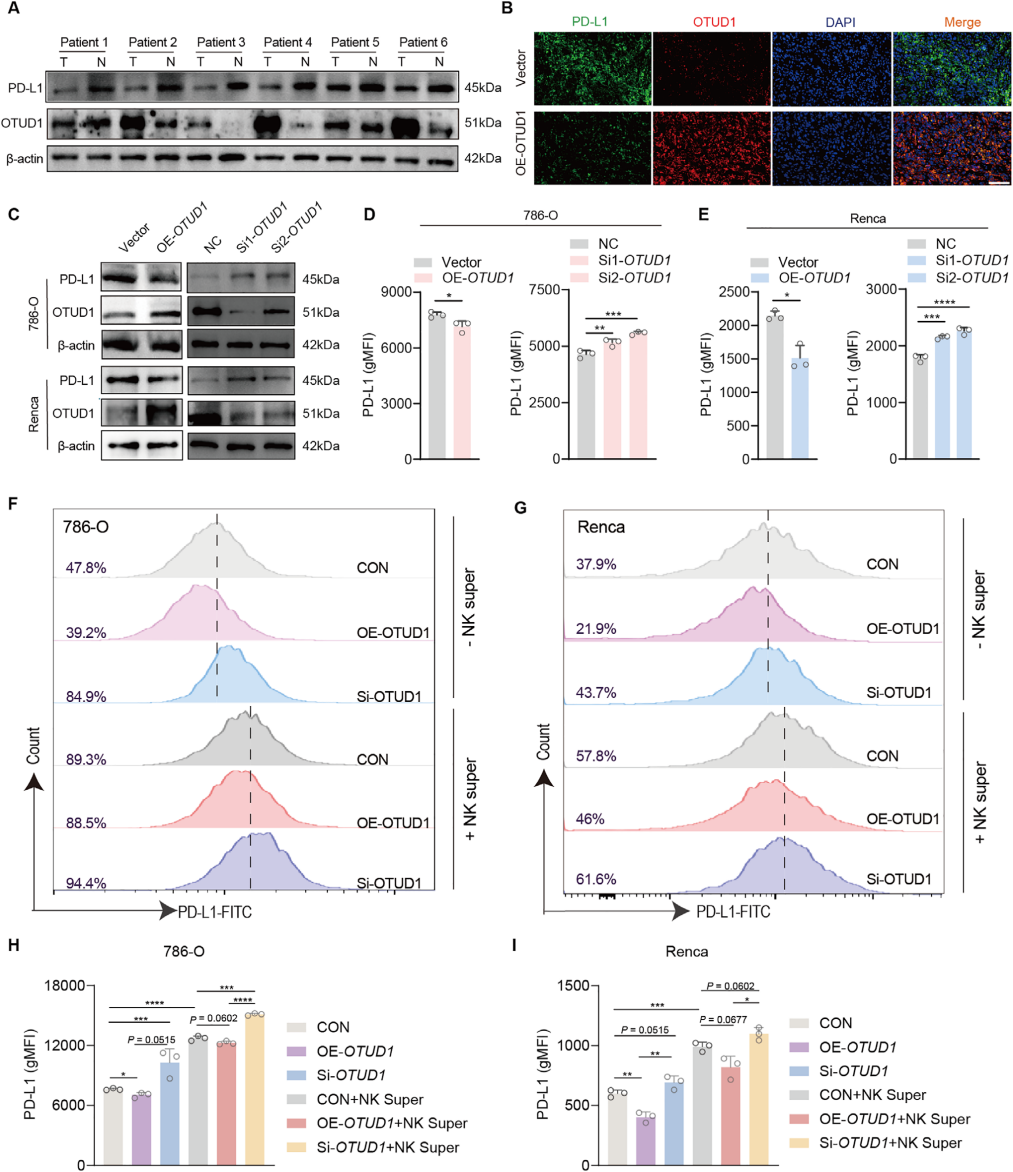
OTUD1 overexpression induced PD‐L1 reduction in KIRC. (A) OTUD1 and PD‐L1 protein expression were detected by Immunoblot analysis for six pairs of randomly selected KIRC tissues and matched para‐cancerous tissues. (B) The collected tumors from 786‐O bearing mouse modelwere analyzed by immunofluorescence staining using antibodies against PD‐L1 (green) and OTUD1 (red). Representative images from two independent experiments are shown. Nuclei were counterstained with DAPI (blue). 200× magnification, Scale bar = 100 µm. (C) Effects of overexpression or knockdown of OTUD1 on PD‐L1 protein expression in 786‐O cells and Renca cells, as detected by Immunoblot analysis. (D) Fluorescence quantitative analysis of flow cytometry for PD‐L1 distribution on the surface of 786‐O cells and (E) Renca cells. The data are expressed as mean ± SD from three biological replicates. (F) Representative flow cytometry analysis for PD‐L1 on the surface of 786‐O cells and (G) Renca cells with or without co‐culture with supernatant of NK cells. (H) Fluorescence quantitative analysis by flow cytometry for PD‐L1 on the surface of 786‐O cells and (I) Renca cells with or without co‐culture with supernatant of NK cells. The data are expressed as mean ± SD from three biological replicates. Significance was assessed using a two‐tailed paired Student's *t*‐test (left panels of D, E), a one‐way ANOVA followed by Dunnett's multiple comparisons test (right panels of D, E; H, I). Data are representative of at least three (A, C) independent experiments with similar results.

### 2.4 OTUD1‐Induced PD‐L1 Reduction Affects T Cell‐Dependent Cytotoxicity in KIRC

PD‐L1 on tumor cells binds to PD‐1 on immune cells, thereby suppressing antitumor immunity, which prompted us to investigate whether OTUD1 influences PD‐L1/PD‐1 interaction. Flow cytometry revealed a significant decrease in the mean fluorescence intensity (gMFI) of membrane PD‐L1 in OTUD1‐overexpressing cells after co‐incubating with a fusion protein containing human/mouse PD‐1 or the Fc fragment of immunoglobulin G (IgG) (Figure S5A–D). To examine whether OTUD1‐mediated PD‐L1 downregulation enhances T cell‐mediated cytotoxicity, we co‐cultured OTUD1‐modulated 786‐O cells with Jurkat T cells and evaluated T cell activation and tumor cell killing (Figure 5A). The gMFI of the activation marker CD69 is increased on Jurkat cells co‐cultured with OTUD1‐overexpressing tumor cells compared with Jurkat cells without co‐culture or co‐cultured with empty‐vector‐transfected tumor cells (Figure 5B,C). To verify whether OTUD1 increases the sensitivity of tumor cells to T cell‐mediated cytolysis by decreasing PD‐L1, we harvested the T cell lysates for mRNA expression by qRT‐PCR and the medium for protein expression by enzyme‐linked immunosorbent assay (ELISA). We observed that OTUD1 significantly increased both the mRNA expression of genes encoding IFN‐γ and tumor necrosis factor (TNF)‐α, but also their secretion in the medium of the co‐culture system (Figure 5D). Apoptosis assays showed a higher rate of tumor cell death upon co‐culture with activated Jurkat T cells in the OTUD1 overexpression group (Figure 5E–F). Taken together, these results indicate that OTUD1 potentiates T cell‐mediated cytotoxicity against tumor cells by destabilizing PD‐L1, thereby likely restoring effector T‐cell functions in the TME of KIRC.

**FIGURE 5:**
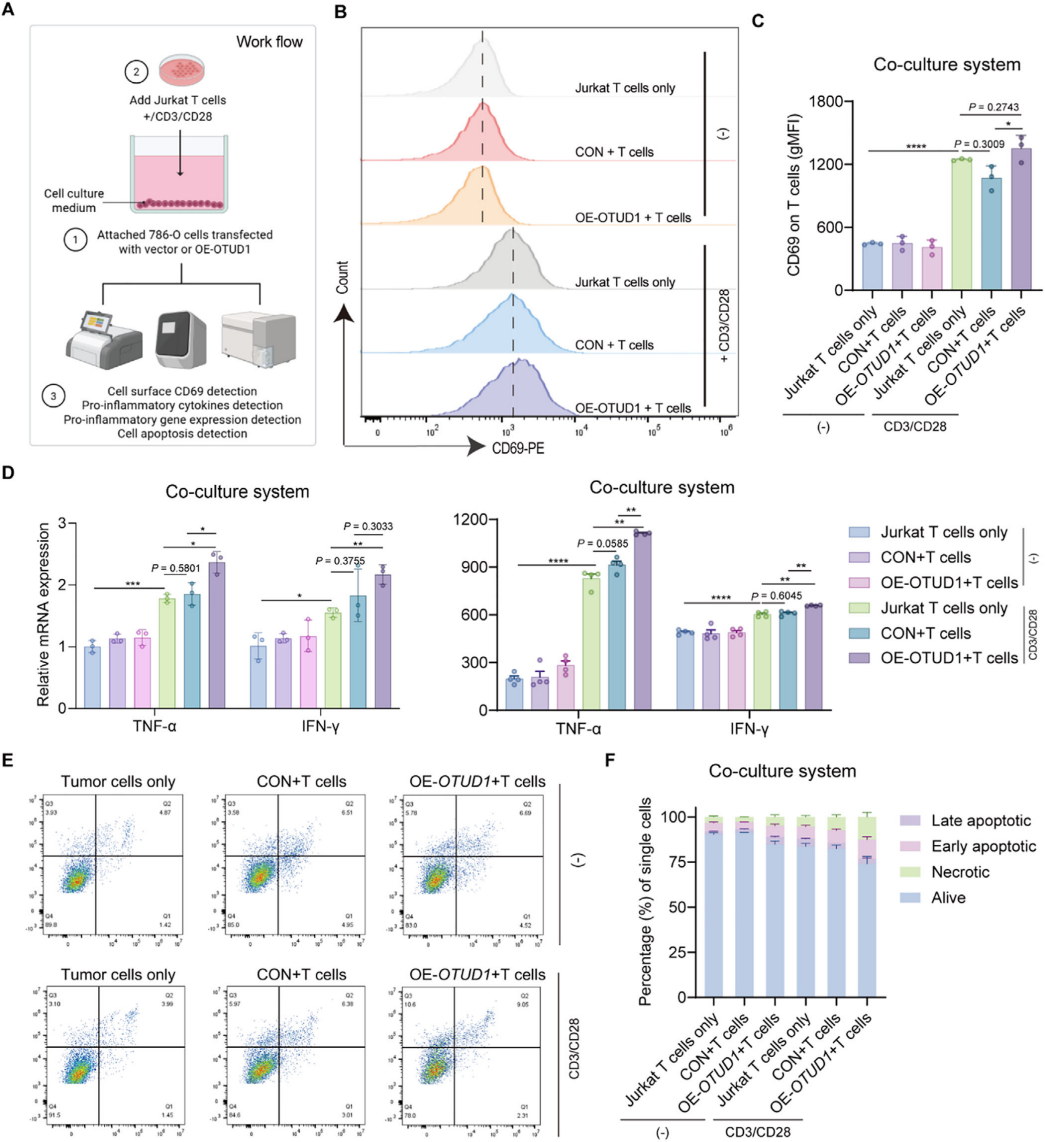
OTUD1‐induced PD‐L1 reduction affects T cell‐dependent cytotoxicity. (A) Schematic representation of the in vitro co‐culture system and subsequent analysis of tumor cells and Jurkat T cells. (B) Representative flow cytometry analysis and (C) statistical graphs of the MFI for CD69 on the surface of T cells, indicating the activation status of the T cells upon co‐culture with tumor cells. The data are expressed as mean ± SD from three biological replicates. (D) Quantitative analysis of the gene expression encoding IFN‐γ and TNF‐α by qRT‐PCR. Relative amount of IFN‐γ and TNF‐α in culture medium by ELISA, following 24 h of Jurkat T cells co‐culture with tumor cells. The data are expressed as mean ± SD from three biological replicates. (E) Representative image of the flow cytometry analysis showed that T cell‐dependent apoptosis occurred in 786‐O cells after co‐culture with Jurkat T cells. (F) The percentages of early apoptotic, late apoptotic, and necrotic cells, as well as live healthy cells, are represented. The data are expressed as mean ± SD from three biological replicates. Significance was assessed using a one‐way ANOVA followed by Tukey's multiple comparisons test (C).

### 2.5 OTUD1 Promotes PD‐L1 Degradation in an Autophagosome‐Lysosomal Pathway

Next, we attempted to investigate how OTUD1 reduces PD‐L1 protein expression in KIRC cells. By half‐life analysis, we investigated the role of OTUD1 in regulating PD‐L1 stability and found that the accelerated degradation of PD‐L1 in OTUD1‐overexpressing 786‐O cells was enhanced by the addition of a protein synthesis inhibitor, cycloheximide (CHX) (Figure 6A,B). Moreover, treatment with the proteasome inhibitor MG‐132 did not rescue the OTUD1‐induced reduction in PD‐L1 (Figure 6C), suggesting that OTUD1‐mediated degradation is proteasome‐independent. Co‐immunoprecipitation and ubiquitination assays further confirmed that OTUD1 neither binds to PD‐L1 nor affects its polyubiquitination status (Figure S6A,B). Therefore, attention was redirected to the OTUD1‐induced degradation of PD‐L1, focusing on the autophagy‐lysosomal pathway. To probe this, autophagy marker proteins and tumor cell morphology were examined. In 786‐O cells, LC3‐II/I was significantly increased after OTUD1 overexpression. At the same time, sequestosome‐1 (SQSTM1)/P62 was notably decreased (Figure 6D). Additionally, transmission electron microscopy (TEM) results revealed increased numbers of autophagosomes and autophagolysosomal structures in OTUD1‐overexpressing cells (Figure 6E). Autophagic flux is considered to be reflective of autophagic degradation activity [24]. Using fluorescent probes DAPRed and DALGreen [25] to monitor autophagic flux, we observed significantly elevated fluorescence intensities in OTUD1‐overexpressing cells corresponding to autophagosomes (DAPRed) and autolysosomes (DALGreen), respectively (Figure 6F,G). Excessive activation of autophagy can trigger the release of danger‐associated molecular patterns (DAMPs), thereby enhancing the immunogenicity of tumor cells [26]. OTUD1 overexpression markedly increased the surface exposure or release of DAMPs, including calreticulin (CRT), ATP, heat shock protein (HSP) 70, and high mobility group protein 1 (HMGB1) in 786‐O cells (Figure S7A–C,G) and Renca cells (Figure S7D–F,G). These effects suggest that OTUD1‐mediated autophagy may contribute to immune activation in KIRC.

**FIGURE 6:**
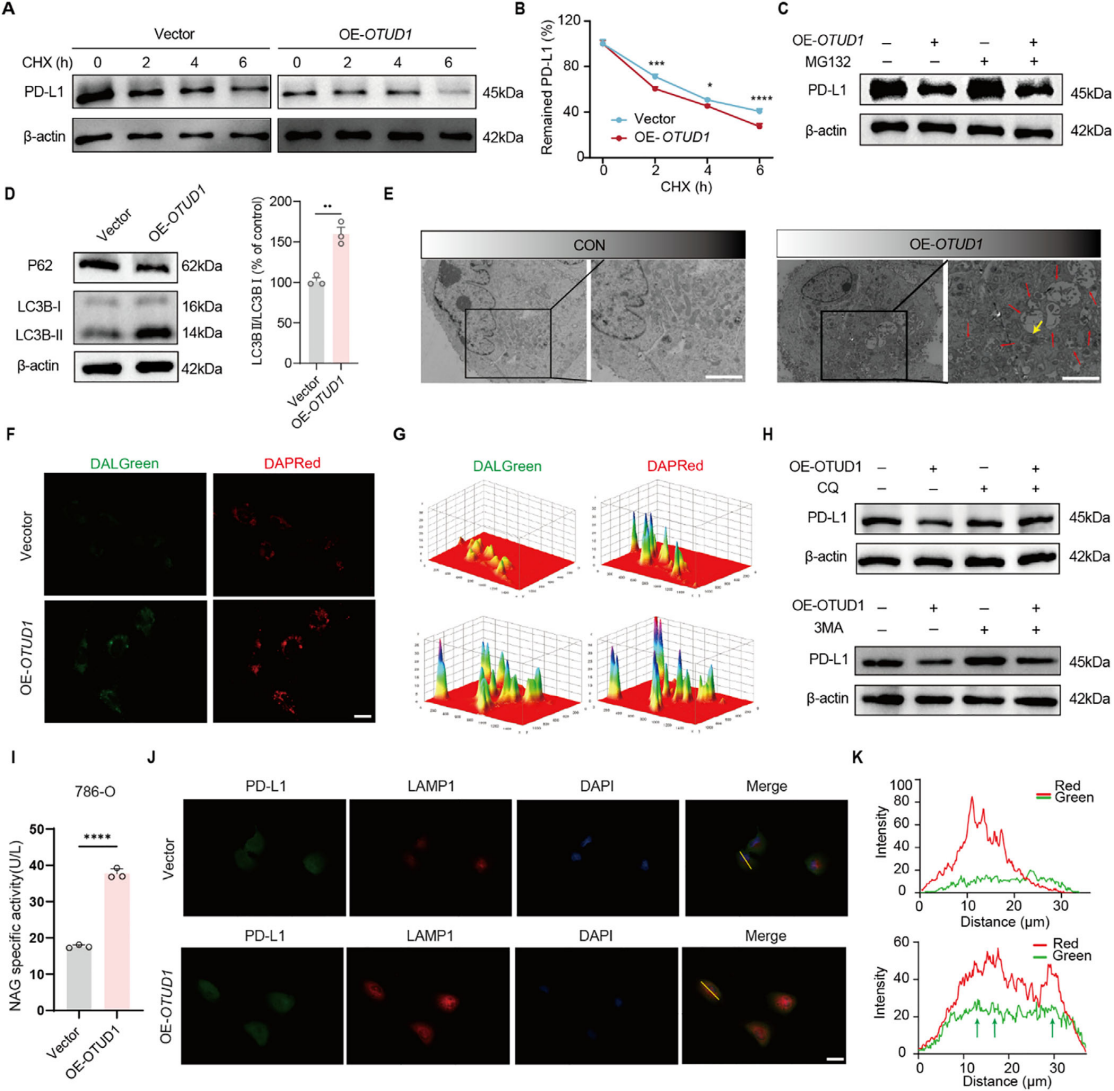
OTUD1 promotes PD‐L1 degradation through the autophagy‐lysosomal pathway. (A) Cycloheximide (CHX) was used to block protein synthesis. Immunoblots of protein expression levels and (B) quantification of PD‐L1 in 786‐O cells overexpressing OTUD1 and control cells. (C) MG132 was used to block proteasome activity. Immunoblots of the protein expression levels of PD‐L1 in 786‐O cells overexpressing OTUD1 and control cells. (D) Immunoblots and quantification of the autophagy‐associated protein expression levels of p62, LC3‐I, and LC3‐II in 786‐O cells overexpressing OTUD1. (E) Transmission electron microscopy (TEM) showing an increase in autophagosomes and autolysosomes in 786‐O cells with OTUD1 overexpression. Representative images from two independent experiments are shown. Yellow arrow: phagosomes; red arrow: phagolysosomes; scale bar = 10 µm. (F) Representative fluorescent microscopy images from two independent experiments and (G) a heat map showing the fluorescence intensity of DALGreen and DAPRed in 786‐O cells with OTUD1 overexpression are shown. Scale bar = 20 µm. (H) CQ and 3MA were used to block the autophagy‐lysosomal pathway. Immunoblots of the protein expression level of PD‐L1 in 786‐O cells overexpressing OTUD1 and control cells. (I) NAG relative activities of 786‐O cells overexpressing OTUD1 and control cells were measured and presented in histograms. The results are expressed as mean ± SD from three biological replicates. (J) Representative fluorescent microscopy images from two independent experiments are shown. (K) The statistical fluorescence intensity showing the localization of PD‐L1 (green) and LAMP1 (red) in 786‐O cells with OTUD1 overexpression and control cells. Scale bar = 20 µm. Significance was assessed using a two‐way ANOVA followed by Šídák's multiple comparisons test (B) and a two‐tailed paired Student's *t*‐test (D, I). Data are representative of at least three (A, C, D, H) independent experiments with similar results.

Then, to determine whether OTUD1 promotes PD‐L1 degradation in an autophagy‐lysosomal manner, 3‐methyladenine (3‐MA) (an autophagosome formation inhibitor) or chloroquine (CQ, a lysosomal acidification inhibitor) was added to OTUD1‐overexpressing cells. Both treatments effectively reversed OTUD1‐mediated PD‐L1 degradation (Figure 6H; Figure S8A,B). We further quantified lysosomal activity using a β‐N‐acetylglucosidase (NAG) assay and LysoTracker staining, which showed a remarkable increase in NAG enzymatic intensity and LysoTracker signal in OTUD1‐overexpressing 786‐O cells compared with the control, reinforcing the conclusion that OTUD1 triggers lysosomal biogenesis and PD‐L1 degradation (Figure 6I; Figure S8C). Notably, previous studies have indicated that a single peptide tyrosine‐based motif (YWHL) enables the lysosomal localization of intracellular PD‐L1 [27]. Similarly, we observed enhanced co‐localization of PD‐L1 with lysosome‐associated membrane protein 1 (LAMP1) in OTUD1‐overexpressing 786‐O cells in contrast to control tumor cells, suggesting OTUD1 promotes the translocation of PD‐L1 toward lysosomes (Figure 6J,K). Taken together, these data demonstrate that OTUD1 enhances autophagosome formation, lysosomal biogenesis and activity, and facilitates the translocation of PD‐L1 to lysosomes for degradation in KIRC cells.

### 2.6 OTUD1 Regulates PD‐L1 Abundance Through the AKT‐mTOR‐TFEB Pathway

To further elucidate the molecular mechanism by which OTUD1 promotes PD‐L1 degradation via the lysosomal pathway, we performed transcriptomic sequencing on 786‐O cells with successful OTUD1 overexpression. Gene Set Enrichment Analysis (GSEA) enrichment analyses of sequencing data identified the mTOR (mammalian target protein of rapamycin) pathway as a candidate of interest due to its well‐established role in autophagy regulation (Figure 7A). As shown in Figure 7B, our results demonstrated that the AKT‐mTOR‐TFEB signaling axis was significantly inhibited upon OTUD1 overexpression, as indicated by diminished phosphorylation levels of AKT, mTOR, and TFEB (transcription factor EB). To determine whether mTOR signaling contributes directly to OTUD1‐mediated PD‐L1 degradation, we added the mTOR‐specific activator MHY1485 to OTUD1‐overexpressing 786‐O cells. Results showed that MHY1485 effectively reversed OTUD1‐induced mTOR suppression, autophagy activation, and partially rescued PD‐L1 degradation (Figure 7C). Canonical mTOR inhibition is known to promote TFEB dephosphorylation and nuclear translocation [28], resulting in the subsequent activation of genes involved in lysosomal biogenesis and autophagy. Thus, we next investigated the subcellular distribution of TFEB. Overexpression of OTUD1 promoted nuclear translocation of TFEB proteins in 786‐O cells, as evidenced by nuclear‐cytoplasmic fractionation assays (Figure 7D). This finding was corroborated by decreased cytoplasmic fluorescence intensity and a corresponding increase in nuclear TFEB fluorescence (Figure 7E). We further assessed the expression of TFEB‐targeted genes involved in autophagy and lysosomal function. The results indicated upregulation of *TPP1*, *MAP1LC3*, *LAMP1*, and *Gabbarap* in OTUD1‐overexpressing 786‐O cells (Figure 7F), consistent with enhanced autophagic flux.

**FIGURE 7:**
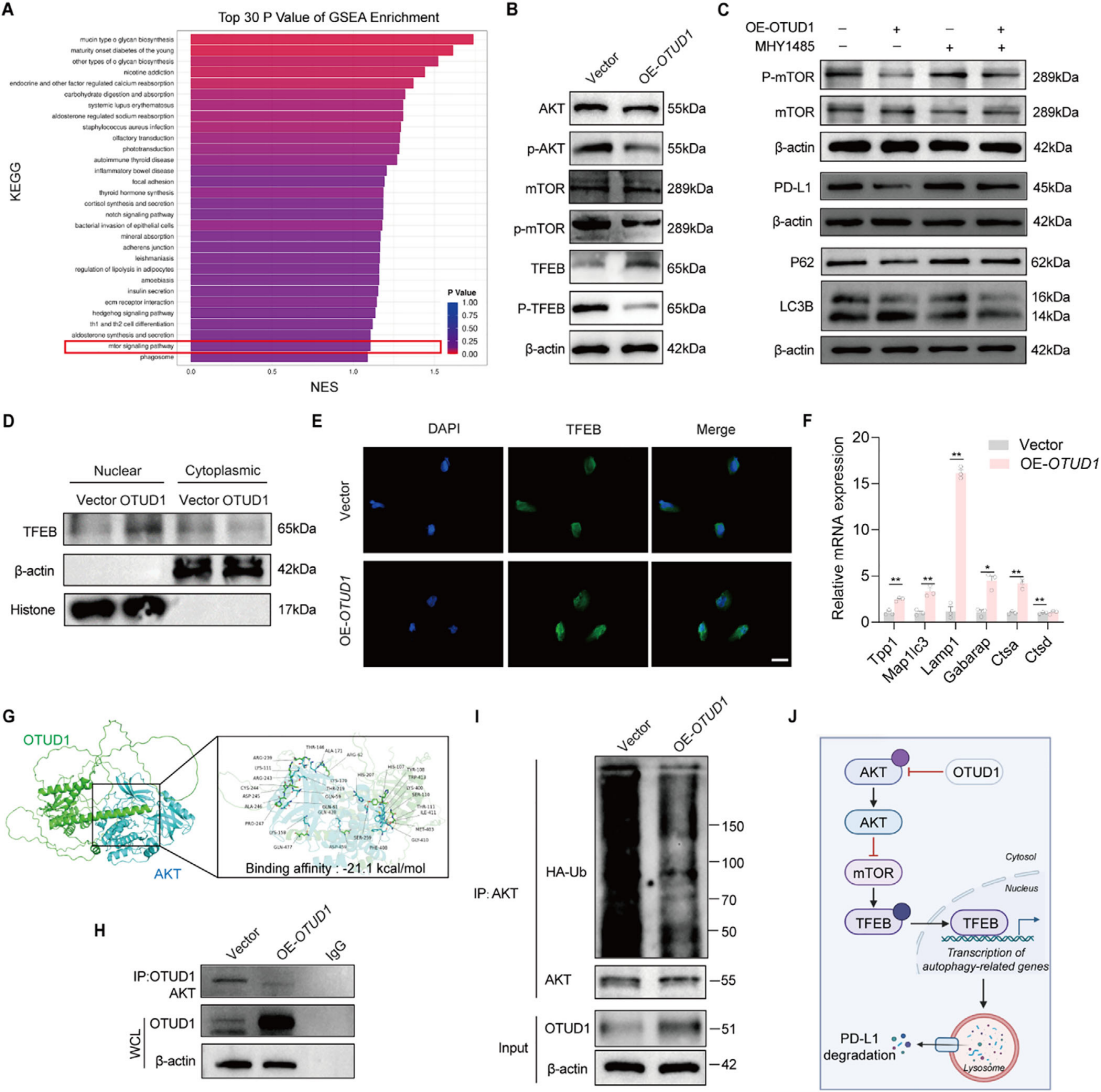
OTUD1 regulates PD‐L1 abundance through the Akt‐mTOR‐TFEB pathway. (A) Transcriptional profiling reveals the key pathways influenced by OTUD1 in 786‐O cells. (B) Immunoblots of the protein expression levels of Akt, p‐Akt, mTOR, p‐mTOR, TFEB, and p‐TFEB in 786‐O cells overexpressing OTUD1 and control cells. (C) MHY1485 was used to activate the mTOR pathway and inhibit autophagy. Immunoblots of the protein expression levels of mTOR, p‐mTOR, P62, LC3B, and PD‐L1 in 786‐O cells with overexpressed OTUD1 expression and control cells. (D) Immunoblots of the protein expression level of cytosolic and nuclear protein accumulation of TFEB in 786‐O cells with overexpressed OTUD1 expression and control cells. (E) Fluorescence microscopy images of TFEB subcellular localization in 786‐O cells with overexpressed OTUD1 expression and control cells. Representative images from two independent experiments are shown. Scale bar = 20 µm. (F) Quantitative analysis of the mRNA expression of *TPP1*, *MAP1LC3*, *LAMP1*, *GABARAP*, *CTSA*, and *CTSD* in 786‐O cells with OTUD1 overexpression and control cells by qRT‐PCR. The results are expressed as mean ± SD from three biological replicates. (G) Molecular docking model of the interactions between OTUD1 and AKT. (H) Co‐immunoprecipitation (Co‐IP) assay was performed to examine the interaction between HA‐OTUD1 and FLAG‐AKT. (I) The effect of AKT ubiquitination by overexpressed OTUD1 was examined in control and OTUD1‐overexpressing 786‐O cells by HA‐Ubiquitin assay. The “vector” group represents cells transfected with the empty plasmid control; the “OE‐OTUD1” group represents cells transfected with the OTUD1‐overexpression plasmid. (J) Schematic working model. OTUD1 binding to AKT decreases mTOR pathway activity, followed by translocation of TFEB to the nucleus, thereby triggering autophagy‐dependent degradation of PD‐L1 in the lysosome. Data are representative of at least three (A, C, D, H, I) independent experiments with similar results.

As previous studies have revealed that OTUD1 binds and inhibits AKT activation, we performed molecular docking using ZDOCK software and visualization analysis via PRODIGY software [29], as well as Co‐IP assay and AKT polyubiquitination assay to verify this finding. Our results suggested that the predicted binding affinity between OTUD1 and AKT was −21.1 kcal/mol, indicating high potential and affinity for complex formation (Figure 7G). Co‐IP assay further validated that exogenously expressed OTUD1 could independently pull down AKT (Figure 7H) and antagonize the polyubiquitination of AKT protein (Figure 7I). Together, these data suggest that OTUD1 inhibits the Akt‐mTOR pathway and facilitates TFEB nuclear translocation, thereby promoting autophagy‐dependent degradation of PD‐L1 (Figure 7J).

## 3 Discussion

OTUD1 has attracted considerable attention due to its critical regulatory roles in cancer progression. Consistent with previous reports [30], our pan‐cancer and KIRC‐specific analyses reveal that OTUD1 is significantly downregulated in tumor tissues and tightly correlates with poor clinical outcomes. Nevertheless, we have not excluded the possibility that OTUD1 has oncogenic effects in specific contexts. For instance, it was reported to promote gastric cancer aggressiveness through deubiquitylation of the Epstein–Barr virus (EBV)‐encoded apoptosis regulatory protein BALF1 via its OTU domain [31], highlighting tumor‐type‐specific functional diversity of DUBs [12]. As a deubiquitinase, OTUD1 elicits intrinsic tumor‐suppressive functions by stabilizing key substrate proteins, such as KLF4 in NSCLC [32] and p53 in ovarian cancer [33]. Aligning with its established role as a tumor suppressor in KIRC [34], our functional data confirm that OTUD1 inhibits KIRC cell proliferation, modulates the cell cycle, and suppresses EMT‐driven invasion, effectively eliminating tumor growth in vivo. Furthermore, OTUD1 suppresses EMT‐driven invasion and migration in KIRC cells, which may be linked to the TGF‐β/SMAD pathway [30].

However, suppression of intrinsic malignant characteristics of tumor cells could not fully explain the profound clinical benefits associated with high OTUD1 expression. KIRC is a highly immunogenic tumor where immune checkpoint‐mediated immunosuppression affects disease progression and therapeutic responses [35]. Our bioinformatic analyses of the TCGA‐KIRC dataset reinforced its immunoregulatory role in KIRC and the striking inverse correlation between OTUD1 and checkpoint molecules (such as CTLA‐4, PD‐1, and PD‐L1). Notably, we found that OTUD1 overexpression increases the infiltration of CD4^+^ and CD8^+^ T cells in KIRC tumors. Findings from Wang et al. [36] suggested that OTUD3, another OTUD family member, acts as a potential negative regulator of PD‐L1 in melanoma by a CRISPR‐based DUB knockout screen. These data prompted us to investigate whether OTUD1 modulated tumor PD‐L1 and immune evasion in KIRC. Indeed, we observed that low OTUD1 expression was associated with high levels of PD‐L1 in both human and murine tumor tissues, suggesting a potential cause‐and‐effect relationship between OTUD1 and PD‐L1 in KIRC. As a result, OTUD1 potentiates T cell‐mediated cytotoxicity against tumor cells by destabilizing PD‐L1, thereby likely restoring effector T‐cell functions in the TME of KIRC. Interestingly, OTUD1 is highly enriched in CD4^+^ T cells and Th17 cells, whereas moderately expressed in CD8^+^ T cells and B cells by analyzing the public single‐cell sequencing dataset (GSE171306) [37] via Uniform Manifold Approximation and Projection (UMAP) dimensionality reduction analysis (Figures S9–S10). Whether OTUD1 regulates antitumor adaptive immunity and humoral immunity in KIRC via intrinsic mechanisms in these immune cells remains unclear and warrants further investigation.

Mechanistically, we showed that OTUD1 regulates posttranslational degradation of PD‐L1, extending the findings by Liang et al. [17] that OTUD1 downregulates PD‐L1 (CD274) at the transcriptional level by deubiquitinating STAT3. Indeed, we found that OTUD1 significantly ameliorates autophagy deficiency and triggers the biogenesis of lysosomes, thereby facilitating PD‐L1 translocation to lysosomes. The more compelling causal evidence suggests that OTUD1‐induced PD‐L1 degradation can be partially reversed by inhibitors of autophagy‐lysosomal enzyme activity, rather than by proteasome inhibition. Commonly, mTORC1 activation on the lysosomal membrane suppressed the activity of TFEB via phosphorylation and cytosolic retention [28]. An exogenous natural compound, Tubeimoside‐1 (TBM‐1), was reported to induce TFEB‐dependent lysosomal degradation of PD‐L1 via mTORC1 inhibition [21]. Here, our data support the viewpoint that OTUD1 controls PD‐L1 protein stability through the AKT‐mTOR‐TFEB‐dependent lysosomal degradation pathway, identifying an endogenous deubiquitinase as the physiological upstream regulator of this precise cascade. Song et al. [15] established that OTUD1 enhances host antitumor immunity by promoting ferroptosis and DAMP release. This inspired us to investigate whether OTUD1 augments broader tumor immunogenicity in KIRC via DAMP signaling beyond PD‐L1 modulation. Our data showed that OTUD1 induces the release of DAMPs (e.g., HMGB1, CRT, ATP, HSP70) in KIRC, which may be an additional immunogenic consequence of OTUD1‐induced activation of autophagy. Notably, the upregulation and cytosolic translocation of HMGB1 are also required for autophagy induction under cellular stress [38], which provides us with further insight into the antitumor mechanisms of OTUD1.

Although the present study did not evaluate combination therapies, high OTUD1 expression represents a promising strategy to overcome resistance to immunotherapy alone. This concept is supported by Liang et al. [17], who demonstrated that OTUD1 deletion attenuates the efficacy of anti‐PD‐1 and reduces infiltration of CD4^+^ and CD8^+^ T cells. In addition, OTUD1 overexpression has been shown to counter resistance to tyrosine kinase inhibitors (TKIs) in KIRC by stabilizing phosphatase and tensin homolog (PTEN), a key negative regulator of the AKT pathway [34]. Consistent with this, treatment with AKT inhibitors has been demonstrated to improve TKI efficacy [39]. Our results confirm that OTUD1 mediates inhibition of the AKT/mTOR pathway and induces cellular autophagy in KIRC. Moreover, a natural bioactive compound (Atractylenolide I) induced autolysosomal degradation of hypoxia‐inducible factor (HIF)2α via the upregulation of ATPase subunit ATP6V0D2, which was shown to overcome sunitinib resistance in KIRC [40]. However, the intricate effects of autophagy in cancer are controversial and complicate the interpretation of the relationship between OTUD1‐induced autophagy and TKI resistance [41]. Thus, the precise contribution of OTUD1‐induced autophagy to TKI sensitivity in KIRC requires more detailed and context‐specific studies.

## 4 Conclusion

In summary, our data demonstrate the clinical significance of OTUD1 in KIRC and confirm its tumor‐suppressive functions both in vitro and in vivo. Importantly, our findings provide novel mechanistic insights into OTUD1‐induced degradation of PD‐L1 through the regulation of the autophagy‐lysosomal pathway. Enhancing the functions of OTUD1 may offer a promising therapeutic strategy to boost antitumor immunity by endogenously downregulating PD‐L1 and increasing tumor immunogenicity. It is noteworthy that OTUD1 participates in essential physiological processes through the regulation of multiple substrates, including oxidative stress, antiviral signaling, and innate and acquired immune responses. Therefore, systemic targeting of OTUD1 is carefully evaluated to avoid on‐target adverse effects and ensure treatment safety. Collectively, this work expands our understanding of OTUD1's biological functions and identifies the OTUD1‐mTOR‐PD‐L1 axis as a critical suppressor of KIRC tumor growth.

## 5 Materials and Methods

### 5.1 Ethical Statement

The study was conducted in accordance with the Declaration of Helsinki principles. Samples were collected from cancer‐oriented biobanks and were approved by the Ethics Committee of China‐Japan Union Hospital of Jilin University. All animal studies were conducted under the guidance of the Animal Welfare and Research Ethics Committee at Jilin University and approved by the Laboratory Animal Center (approval number: SY202410002, Changchun, China).

### 5.2 Cell Lines and Cell Culture

786‐O, stable OTUD1‐overexpressing 786‐O, Renca, and stable OTUD1‐overexpressing Renca cell lines were obtained from Applied Biological Materials (abm, Jiangsu, China) and were checked for authenticity by short tandem repeat (STR) profiling. 786‐O and Renca were cultured in RPMI 1640 (abm Cat. No. TM503) supplemented with 10% fetal bovine serum (FBS, abm Cat. No. TM999) and 1% Penicillin/Streptomycin (abm Cat. No. G255). Stable OTUD1‐overexpressing 786‐O and Renca cell lines were cultured in RPMI 1640 supplemented with 10% FBS, 1% penicillin/streptomycin G255), and 0.7 µg/mL puromycin. All cell lines are maintained in a humidified incubator at 37°C in 5% CO_2_ and regularly tested for the absence of mycoplasma via PCR.

### 5.3 Antibodies, Chemicals, and Plasmids

The antibodies used in this study are listed in Table S1. Cycloheximide (CHX), MHY1485, chloroquine (CQ), and 3‐Methyladenine (3MA) were products of MedChemExpress (MCE, USA). The plasmids pCMV‐T7‐MCS‐3×FLAG‐WPRE‐Neo and pCMV‐OTUD1‐3×FLAG‐Neo are from MIAOLING PLASMID (China).

### 5.4 SiRNA Transfection

The siRNA oligonucleotides targeting human/mouse si‐OTUD1 and a nontargeting negative control siRNA (si‐NC) were purchased from Genscript Biotech (Nanjing, China). 786‐O cells and Renca cells were seeded in six‐well plates at an optimized density to reach approximately 60% confluence at the time of transfection. Synthetic siRNAs targeting human OTUD1 (siOTUD1‐1299: 5′‐CAGAGGAAUAAGUACCGAA‐3′; siOTUD1‐1596: 5′‐GUGUCUACCAUGAUUCAUU‐3′) or mouse OTUD1 (siOtud1‐975: 5′‐CCACUUCAGCCCACUCAUU‐3′; siOTUD1‐1046: 5′‐CCGGAUAUCCCGAAUUGCU‐3′), as well as a nontargeting scrambled control siRNA (siNC), were synthesized by Genscript Biotech. Transfections were performed using the and transfected using (ThermoFisher, Cat: L3000015) according to the manufacturer's protocol. The siRNA was diluted in 8.5 µL of Buffer to a final concentration of 1.5 µM, followed by addition of 1.5 µL of Plus reagent. The mixture was vortexed vigorously for 30 s to form siRNA/Plus complexes and incubated at room temperature for 15 min. The complex was then added directly to cells in complete growth medium. After 6 h, the medium was replaced with fresh complete medium. Gene knockdown efficiency was assessed at 24 h posttransfection by quantitative real‐time PCR (qRT‐PCR) for mRNA levels, and at 48 h for protein expression by Western blotting.

### 5.5 RNA Isolation and RT‐qPCR Assays

Unizol Total RNA Extraction Reagent (Genesand Biotech Co, Cat: RE703) was used to extract the total RNA of cells and cancer tissues in accordance with its protocol. The concentration and quality of the extracted RNA were determined by a NanoDrop 2000 Spectrophotometer (Thermo Fisher Scientific). Subsequently, total RNA was reverse transcribed to cDNA using a UnionScript First‐strand cDNA Synthesis Mix for qPCR (Genesand Biotech Co, Cat: SR511). Quantitative PCR (qPCR) was performed using the GS AntiQ SYBR Green Fast Mix Kit (Genesand Biotech Co, Cat: SQ410). The cycling conditions were as follows: 95°C for 15 min followed by 40 cycles of 95°C for 15 s, 60°C for 30 s, and 70°C for 20 s. The relative gene expression levels were calculated by the 2^–ΔΔCt^ method. β‐Actin was used as an internal control. Each assay was performed in triplicate, and representative results are shown. The sequences of qPCR primers can be found in Table S2.

### 5.6 Western Blot Analysis

Cells were washed with cold phosphate‐buffered saline (PBS) and then lysed with radioimmunoprecipitation assay (RIPA, MA0151, Meilunbio, China) buffer supplemented with protease inhibitors (MB2678, Meilunbio) and phosphatase inhibitors (GRF102, Epizyme, China). The extraction of total proteins used the BCA protein assay kit (KTD3001, Abbkine, China) for quantification. Equal amounts of lysates mixed with loading buffer were separated by SDS‐polyacrylamide gel electrophoresis (PAGE) (Omni‐Easy, Bis‐Tris, 6%−15%; PG210) and transferred to 0.22 or 0.45 µm PVDF membrane (Merck Millipore, Cat: ISEQ00010, Cat: IPVH00010). The proteins were then probed with specific primary antibodies followed by horseradish peroxidase (HRP)‐conjugated secondary antibodies (ZenBio, 511103, 1:10,000; ZenBio, 511203, 1:10,000). After incubation with the primary and secondary antibodies, the substrate membrane was incubated in ECL solution (Abbkine, Cat: BMU102‐CN) for 2–3 min. Western blot images were captured by an Image Quant LAS4000 chemiluminescence imaging system (GE Healthcare), and the proteins of interest were quantified using ImageJ (NIH, version 1.53t). All experiments were performed at least three times, and representative results are shown. The detailed information on the antibodies and reagents used in Western blotting is provided in Table S1, and the original and uncropped films of Western blots in this study can be found in Figures S11–S18.

### 5.7 Co‐Immunoprecipitation (Co‐IP) and Ubiquitination Assays

A total of 1 × 10^5^ ccRCC cells were seeded in a 6 cm dish and then transfected with plasmids using Lipofectamine 3000 (ThermoFisher, Cat: L3000015) the following day. Forty‐eight hours after transfection, MG132 (20 µM) was added to treat the cells for 4 h. Each dish of cells was lysed on ice with 500 µL of lysis buffer (50 mM Tris‐HCl pH 7.4, 1 mM EDTA, 1% Triton X‐100, 150 mM NaCl) with Protease Inhibitor Cocktail for 30 min. The protein lysate was divided into two equal portions: one portion was for co‐IP, and the other portion was for ubiquitination IP. The portion for ubiquitination IP was further lysed on ice for 15 min following the addition of 10 µL of 20% (w/v) SDS, and then boiled for 15 min to denature. After centrifugation at 1.2 × 10^4^ rpm for 10 min at 4°C, the supernatant of the portion for normal co‐IP was collected in a new 1.5 mL tube. Some protein samples were prepared as the Input control. The remaining protein sample was incubated with anti‐Flag (MedChemExpress, Cat: HY‐K0207) or anti‐HA (MedChemExpress, Cat: HY‐K0237) magnetic beads at 4°C for 4–6 h. The immunoprecipitation (IP) complexes were washed with wash buffer (50 mM Tris‐HCl pH 7.4, 150 mM NaCl, 0.1% NP‐40, 2 mM EDTA pH 8.0) five times, each lasting 5 min with shaking at 4°C. Each sample was added with 40 µL of 1 × loading buffer and heated at 98°C for 10 min to denature. The IP samples were analyzed by Western blotting (WB) using a 10% SDS‐PAGE gel, and the samples of ubiquitination IP were analyzed by a 6% SDS‐PAGE gel.

### 5.8 Cell Cycle and Apoptosis Analysis by Flow Cytometry

Each well of a six‐well plate was seeded with 1.0 × 10^5^ 786‐O cells. The next day, cells were transfected with siRNA and overexpression plasmids for 24 h using Lipofectamine 3000. For cell cycle analysis, cells were collected and fixed with 75% ethanol for 30 min at room temperature and then washed twice with PBS and resuspended in 200 µL of PBS containing 3 × 10^5^ cells. To treat the cells, 0.5 µL of 10 mg/mL RNase A (Beyotime, Cat: ST576) was added, followed by the addition of 5 µL of 10 mg/mL propidium iodide (PI) (Beyotime, Cat: ST1569) for staining in the dark for 30 min. For apoptosis analysis, cells were harvested and stained with Annexin V‐FITC and PI according to the manufacturer's instructions (Annexin V‐FITC Apoptosis Detection Kit, Beyotime, C1062S). The samples were analyzed by flow cytometry (Beckman Coulter Cytoflex), and data were analyzed using CytExpert V2.3 and FlowJo V10 software.

### 5.9 Cell Invasion and Migration Assays

Each well of a six‐well plate was seeded with 1.0×10^5^ 786‐O cells. The next day, cells were transfected with siRNA and overexpression plasmids for 24 h using Lipofectamine 3000. 786‐O cells at the logarithmic growth phase were digested and transferred to a centrifuge tube. The supernatant was discarded after centrifugation (800 rpm, 5 min), and cells were resuspended in 1 mL of RPMI 1640 medium (serum‐free) followed by cell counting. 2 × 10^5^ cells resuspended in 200 µL serum‐free RPMI 1640 medium were then seeded in the Transwell chamber (8 µm pore size, Corning); 500 µL RPMI 1640 medium containing 20% FBS was added to the bottom of 24‐well plates to induce cell migration. 100 µL of 1:8 diluted Matrigel (Corning, Cat: 356234) was spread in the Transwell chamber 3 h in advance and placed in the incubator at 37°C to solidify. After incubating for 24 h, the culture inserts were fixed with 4% paraformaldehyde for 15 min and stained with 0.1% crystal violet. Cells that stayed on the top of the membrane were gently scraped with a cotton swab. Cells were cultured in 1% serum RPMI 1640 medium and placed in a humidified 5% CO_2_ incubator at 37°C for the indicated time points, and images were taken by phase‐contrast microscopy. Each assay was triplicated.

### 5.10 RNA‐seq Analysis

The total RNA of 786‐O cells was extracted using TRIzol reagent (Invitrogen) and further subjected to RNA‐seq analysis. All reads were uniquely mapped to a gene to evaluate gene expression levels. The dysregulated genes (fold change ≥1.5, *p*  <  0.05) further underwent GO/KEGG enrichment analysis.

### 5.11 Flow Cytometry Analysis of Membrane PD‐L1 and PD‐L1/PD‐1 Binding Ability

For detection of membrane PD‐L1, 786‐O and Renca cells were collected by centrifugation at 800 rpm for 5 min and blocked with PBS containing 0.5% bovine serum albumin for 10 min at room temperature. The cells were then stained with a Fluor 488‐conjugated anti‐PD‐L1 antibody or a matched isotype control at 4°C for 30 min in the dark. To assess unbound PD‐1, cells were first incubated with a fusion protein containing human/mouse PD‐1 or the Fc fragment of immunoglobulin G (IgG) at 4°C for 1 h, followed by staining with Fluor 488‐conjugated anti‐PD‐L1. After staining, cells were washed three times with PBS and resuspended for analysis. Flow cytometry was performed using a Beckman Coulter Cytoflex instrument, and data were processed with CytExpert V2.3 and FlowJo V10 software. The median fluorescence intensity (MFI) of unbound PD‐L1 was used to evaluate the binding ability of PD‐L1 with PD‐1.

### 5.12 T‐Cell Activation Induction

786‐O cells (2.5 × 10^4^) were seeded and cultured in 24‐well plates in RPMI 1640 supplemented with 10% FBS for 24 h. Cells were then transfected with empty or OTUD1‐expressing vectors for 24 h, and the culture medium was changed to RPMI 1640 supplemented with 10% FBS and 1% penicillin/streptomycin. After incubating tumor cells for another 24 h, unstimulated or activated Jurkat T cells (2.5 × 10^5^) were added directly to tumor cells. For the activation of Jurkat T cells, cells were cultured in medium containing 2 µg/mL anti‐human CD3 mAb (Yeasen, Cat: 32101ES60), 1 µg/mL soluble anti‐human CD28 mAb (Yeasen, Cat: 32131ES60), and 5 µg/mL purified mouse IgG2a (Yeasen, Cat: 32283ES60). Twenty‐four hours after co‐culture, the medium in the co‐culture system was harvested for lFN‐γ/TNF‐α ELISA assay; cells were harvested for CD69 (Proteintech, Cat: PE‐98173) staining and analyzed by flow cytometry. Following 24 h co‐culture of tumor cells and T cells, T‐cell cytotoxicity was assessed via apoptotic flow cytometry.

### 5.13 Lysosome Activity Assessment by β‐N‐Acetylglucosaminidase (NAG) Assay and LysoTracker Staining

To assess the effects of OTUD1 overexpression on lysosomal activity, we performed the NAG assay and LysoTracker staining. The activity of NAG was determined in 786‐O cell lysates using the β‐N‐acetylglucosaminidase (NAG) assay kit (Nanjing Jiancheng Bioengineering Institute, Cat: A031‐1‐1) according to the manufacturer's instructions. The absorbance of all lysates was measured at 400 nm, and NAG activity was calculated based on the amount of p‐nitrophenol released. LysoTracker Green DND‐26 (MeilunBio, Cat: MB6042) was used to label the lysosomes at 37°C for 30 min. The medium was aspirated and washed two times with PBS quickly so that the unbound dye can be removed. Green fluorescence was detected using a fluorescence microscope (Olympus).

### 5.14 Immunofluorescent Staining

786‐O cells in logarithmic growth phase were digested and seeded onto coverslips in 24‐well plates. Cells were transfected with either an empty vector or an OTUD1‐overexpressing plasmid using Lipofectamine 3000 according to the manufacturer's instructions. After 48 h of transfection, cells were fixed with 4% paraformaldehyde at room temperature for 15 min, followed by permeabilization with 0.5% Triton X‐100 for 10 min. After three washes with PBS, nonspecific binding was blocked by incubation with 5% goat serum at room temperature for 1 h. For LAMP1 and PD‐L1 co‐localization analysis, cells were incubated overnight at 4°C with primary antibodies against LAMP1 (1:400, Proteintech, Cat: 84658‐5‐RR0) and PD‐L1 (1:400, Proteintech, Cat: 66248‐1‐Ig). For HMGB1 and TFEB immunofluorescence staining, cells were incubated with anti‐HMGB1 antibody (1:200, Proteintech, Cat: 10829‐1‐AP) or anti‐TFEB antibody (1:500, ZenBio, Cat: R27339) under the same conditions. Following PBS washes, cells were incubated with Alexa Fluor 488‐ and Alexa Fluor 594‐conjugated secondary antibodies (1:200, Invitrogen) in the dark for 1 h at room temperature. Nuclei were counterstained with DAPI (1:100, Beyotime, Cat: C1002) for 5 min. Coverslips were mounted with antifade mounting medium (Servicebio, Cat: G1401‐5ML), and images were acquired using a fluorescence microscope (Olympus). Fluorescence signals were analyzed, and co‐localization was assessed using ImageJ software.

### 5.15 ATP Assay

To measure the release of ATP from treated cancer cells, the supernatant from each group was collected. The concentration of ATP was quantified using an ATP assay kit (Beyotime, Cat: S0026) according to the manufacturer's protocols on a microplate reader (Infinite 200 PRO, TECAN).

### 5.16 Cell Counting Kit‐8 Assays

Cell counting kit‐8 (CCK‐8) assays were performed to assess cell proliferation. Following transfection, 786‐O cells were plated in 96‐well plates at a density of 3000 cells per well and incubated at 37°C for 24 and 48 h. CCK‐8 reagent (MedChemExpress, Cat: HY‐K0301) diluted 1:10 with RPMI 1640 medium was added and incubated at 37 °C for 1–2 h. The absorbance value of each well at OD450nm was measured using a microplate reader.

### 5.17 Nuclear/Cytoplasmic Protein Extraction

Nuclear proteins were isolated from cells using a nuclear protein extraction kit (Beyotime, Cat: P0027), following the manufacturer's instructions. Briefly, cells were transfected for 24 h and washed once with cold PBS. Samples were centrifuged at 800 rpm for 5 min. Then, they were resuspended in cytosolic protein extraction reagent A and incubated on ice for 15 min to lyse cell membranes. Cytosolic protein extraction reagent B was added, followed by centrifugation at 12,000×*g* for 15 min. The supernatant contained cytoplasmic protein. The remaining fraction was resuspended with nucleoprotein extraction reagent, incubated on ice for 30 min, and centrifuged at 12,000×*g* for 15 min. The supernatant contained extracted nucleoprotein. Protein concentration was determined using the BCA Kit (Abbkine, Cat: KTD3001).

### 5.18 Xenograft Tumors

Five‐week‐old female BALB/c mice or nude mice were obtained from Beijing Vital River Laboratory Animal Technology Co., Ltd. and housed under specific pathogen‐free (SPF) conditions. Animals were randomly allocated to receive a subcutaneous injection of 100 µL containing 1.0 × 10^7^ cells of either Renca, 786‐O or OTUD1‐overexpressing cells suspended in 1 × PBS. Tumor development was monitored regularly for 40 days postinoculation, after which mice were euthanized. Tumor weight and maximal cross‐sectional area were quantified. Additionally, mice were monitored for survival and euthanized according to the humane endpoints established in the code of practice for cancer research, including accelerated tumor growth exceeding 20 mm; body weight loss of 15% or more in comparison to its start weight or previous measurement; and other signs of distress. Tumor tissues were fixed in 4% paraformaldehyde for following dehydration, embedding, and preparation of paraffin sections. Paraffin‐embedded tissues were subjected to hematoxylin and eosin (H&E) staining for histopathological evaluation and immunohistochemical staining for Ki‐67 to assess tumor cell proliferation.

### 5.19 Statistical Analysis

All data were analyzed using a two‐tailed unpaired Student's *t*‐test, or one‐/two‐way ANOVA with GraphPad Prism software (version 10.2.3, La Jolla, CA, USA). Data are presented as mean ± standard deviation (SD) from three independent biological replicates. Western blotting, immunohistochemical, and immunofluorescence images are representative of experiments repeated independently two or three times with consistent results. The exact value of n is indicated in the figure legends to represent the number of technical or biological replicates used in the experiments. Differences were considered statistically significant at *p* < 0.05. Significance is denoted as follows: *p < 0.05, **p < 0.01, ***p < 0.001, ****p < 0.0001.

## Author Contributions

Conceptualization: H.Y.; data collection: Z.H.Z. and L.T.T.; writing – original draft preparation: H.Y. and Z.H.Z.; writing – review and editing: L.Y.X., F.Q., S.T., J.M.J., L.C.H., L.Q.R., and Z.Y.H.; figure – original draft preparation: H.Y. and Z.H.Z.; figure – visualization: H.Y. and Z.H.Z.; supervision: H.Y. and W.D.X.; final editing: H.Y. All authors have read and approved the final manuscript.

## Funding

This research was supported by the National Natural Science Foundation of China (No. 8250116302).

## Ethics Statement

The study was conducted in accordance with the principles outlined in the Declaration of Helsinki. Samples were collected from cancer‐oriented biobanks and were approved by the Ethics Committee of the China‐Japan Union Hospital of Jilin University (No. 2024‐KYYS‐119). Written consent was obtained from all participants prior to their donation of samples. All animal studies were conducted under the guidance of the Animal Welfare and Research Ethics Committee at Jilin University and approved by the Laboratory Animal Center (No. SY202410002).

## Conflicts of Interest

The authors declare no conflicts of interest.

## Supporting information



Supporting File 1: mco271144‐sup‐0001‐SuppMat.pdf

## Data Availability

The data that support the findings of this study are presented within the article and the Supporting Information files.
