# Supplementary material for: OTUD1 Prevents Immunosuppression via Promoting Lysosomal‐Associated Degradation of PD‐L1
Source: MedComm (2020). 2026 Nov 8;7(11):e71144. doi: 10.1002/mco2.71144 (PMC13744645; doi:10.1002/mco2.71144)
Supplement: Supplementary file 1 [file MCO2-7-e71144-s001.pdf]

OTUD1 prevents immunosuppression via promoting lysosomal-associated degradation of PD-L1

Yang Hao[a], Hengzong Zhou[a], Yanxin Liu[b], Minjun Jin[a], Qiang Feng[a], Tiantian Li[a], Qirong Li[a], Yahui Zhen[a], Tong Sun[a, c], Chenhao Li[d], Dongxu Wang[a], *

a Laboratory Animal Center, College of Animal Science, Jilin University, Changchun, China

b Department of Gastric and Colorectal Surgery, General Surgery Center, The First Hospital of Jilin University, Changchun, China.

c Department of Breast Surgery, China-Japan Union Hospital of Jilin University, 130033, Changchun, Jilin, China.

d Department of Nephrology, Lequn Branch, The First Hospital of Jilin University, Changchun, Jilin, China.

* Corresponding author. Laboratory Animal Center, College of Animal Sciences, Jilin University, Changchun, 130062, PR China.
wang_dong_xu@jlu.edu.cn (Dongxu Wang)

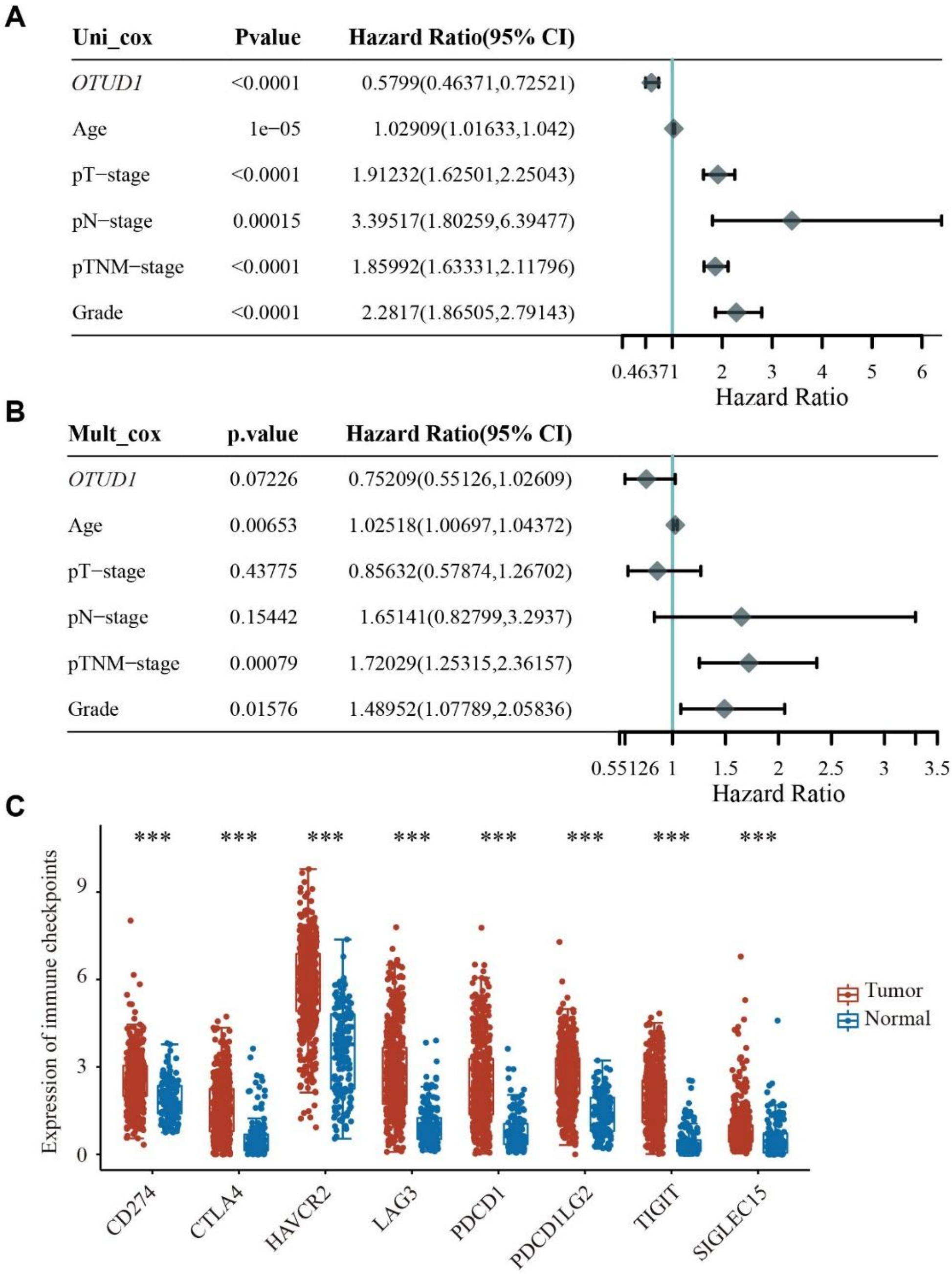

Figure S1 ***OTUD1*** **expression in various KIRC cell lines and it has diagnostic and prognostic value for KIRC patients.** (A) Each individual normalized transcript per million (nTPM) of *OTUD1* in each KIRC cell line analyzed via XIANTAO tool (https://www.xiantao.love/). Cell lines derived from metastatic sites are indicated in orange color, cell lines are derived from *in situ* sites indicated in orange color, cell lines without a clearly defined origin tissue are marked in black. (B) Univariate and (C) multivariate survival analyses for the indicated prognostic factors. pT, primary tumor; pN, regional lymph nodes status; pTNM, tumor-node-metastasis systems.

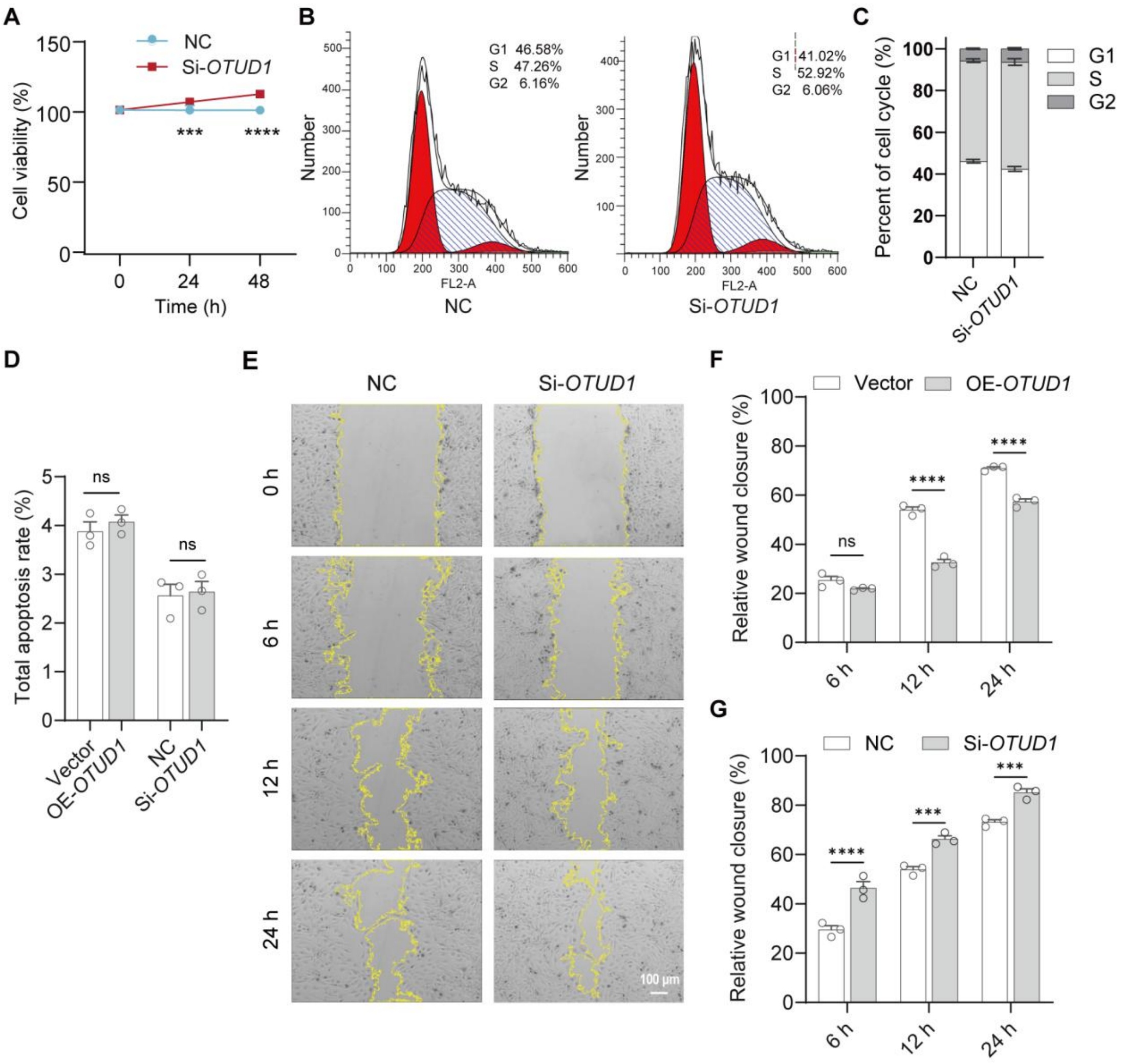

Figure S2 OTUD1 suppression promotes cancer progression in KIRC. (A) Effects of knockdown of *OTUD1* on 786-O cell viability, as measured by Cell Counting Kit-8 (CCK-8) assays. (B) Representative flow cytometry analysis and (C) quantitative analysis for cell cycle distribution of 786-O cells. (D) Percentages of apoptotic cells in 786-O cells with ectopic expression or deletion of OTUD1 were revealed by histograms. The data are expressed as mean ± SD from three biological replicates. (E) The Transwell cell migration assay revealed the effects of knocking down OTUD1 on cell migration. Representative images from two independent experiments are shown. Scale bars = 100 μm. (F) Quantitative analysis for migration ability of 786-O cells with overexpression or (G) knockdown of OTUD1. Significance was assessed using a two-way ANOVA followed by Šídák's multiple comparisons test (F, G). The data are expressed as mean ± SD from three biological replicates.

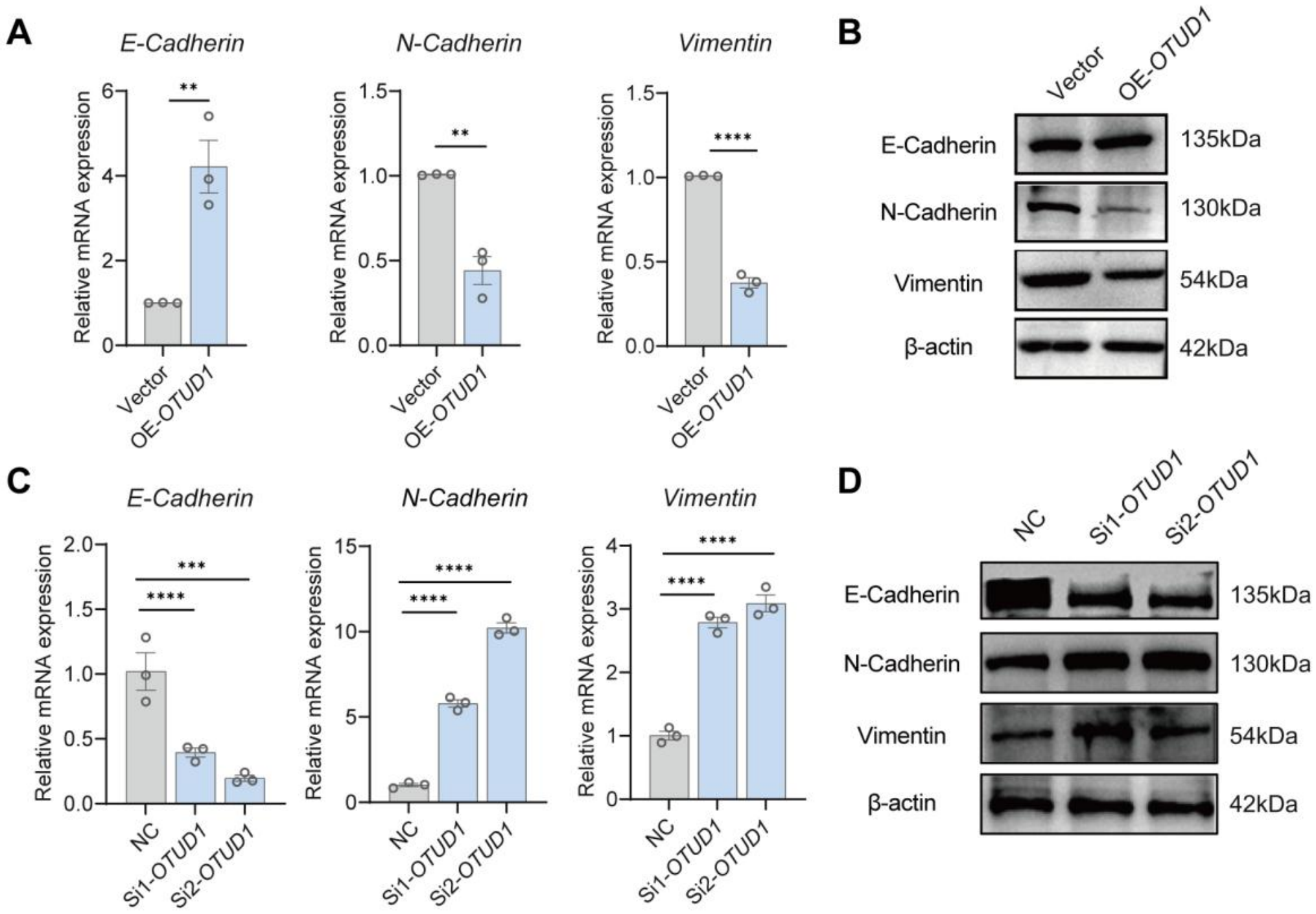

Figure S3 Effects of OTUD1 expression on Epithelial-Mesenchymal Transition (EMT) markers. (A) RT-qPCR analysis and (B) Western Blot analysis of the effects of OTUD1 overexpression on OTUD1, E-cadherin, N-cadherin, and Vimentin mRNA expression in 786-O cells. (C) RT-qPCR analysis and (D) Western Blot analysis of the effects of OTUD1 knockdown on genes encoding OTUD1, E-cadherin, N-cadherin, and Vimentin mRNA expression in 786-O cells. The data are expressed as mean ± SD from three biological replicates (A, C). Significance was assessed using a two-tailed paired Student's t-test (A, C). Data are representative of at least three independent experiments with similar results (B, D).

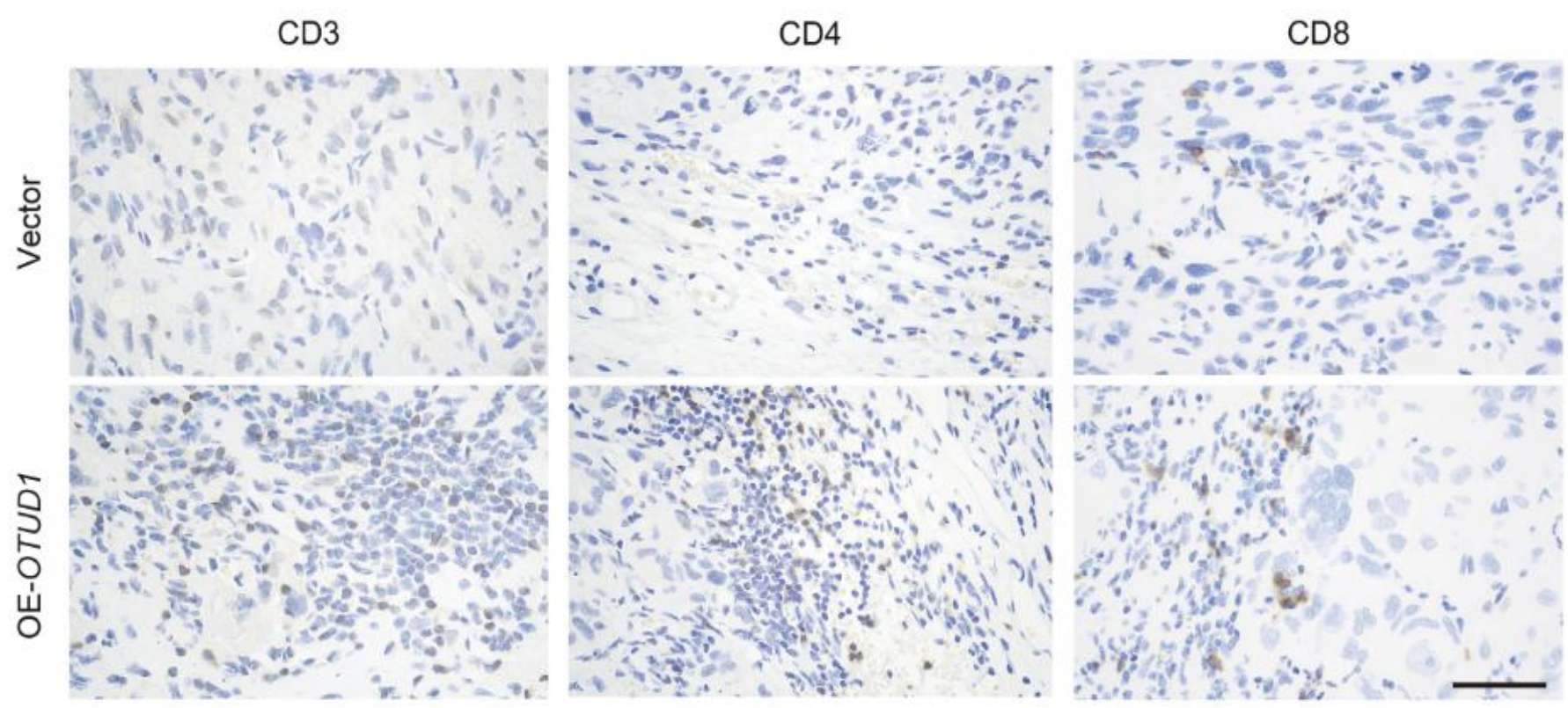

Figure S4 Representative immunohistochemistry (IHC) staining images showing that overexpression of OTUD1 associated with higher CD3, CD4, and CD8 expression in tumors. Scale bar = 20 μm. Representative images are shown at 400× magnification, scale bar = 100 μm.

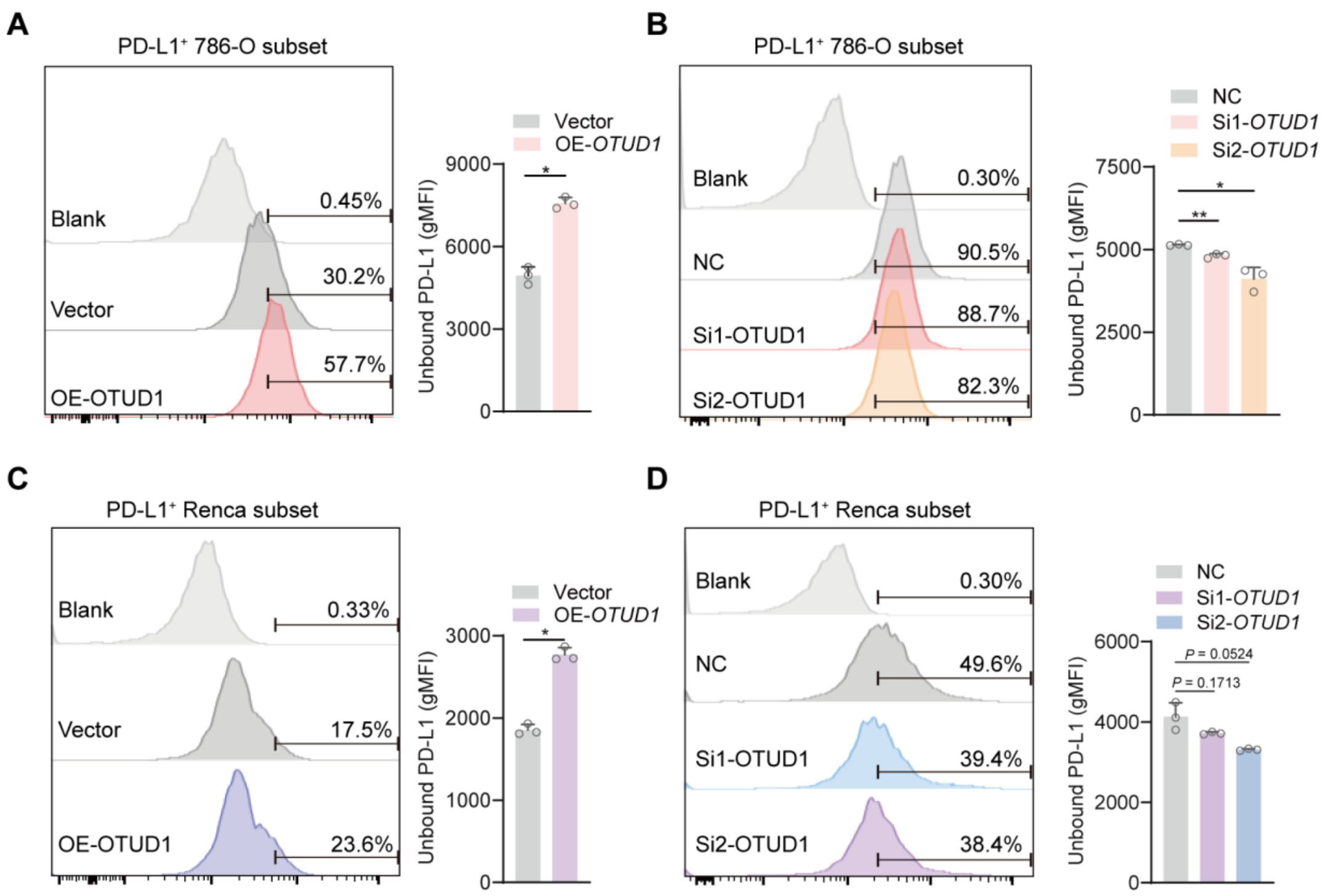

Figure S5 OTUD1 overexpression decreases PD-L1/PD-1 binding ability. (A) Representative flow cytometry analysis for unbound PD-L1 in OTUD1-overexpressing 786-O cells and (B) OTUD1-knockdown 786-O cells after co-incubating with a fusion protein containing human/mouse PD-1 or the Fc fragment of immunoglobulin G (IgG). (C) Representative flow cytometry analysis for unbound PD-L1 in OTUD1-overexpressing Renca cells and (D) OTUD1-knockdown Renca cells after co-incubating with a fusion protein containing human/mouse PD-1 or the Fc fragment of IgG. The data are expressed as mean ± SD from three biological replicates and analyzed using a two-tailed paired Student's *t*-test (A-D). gMFI: Geometric mean fluorescence intensity.

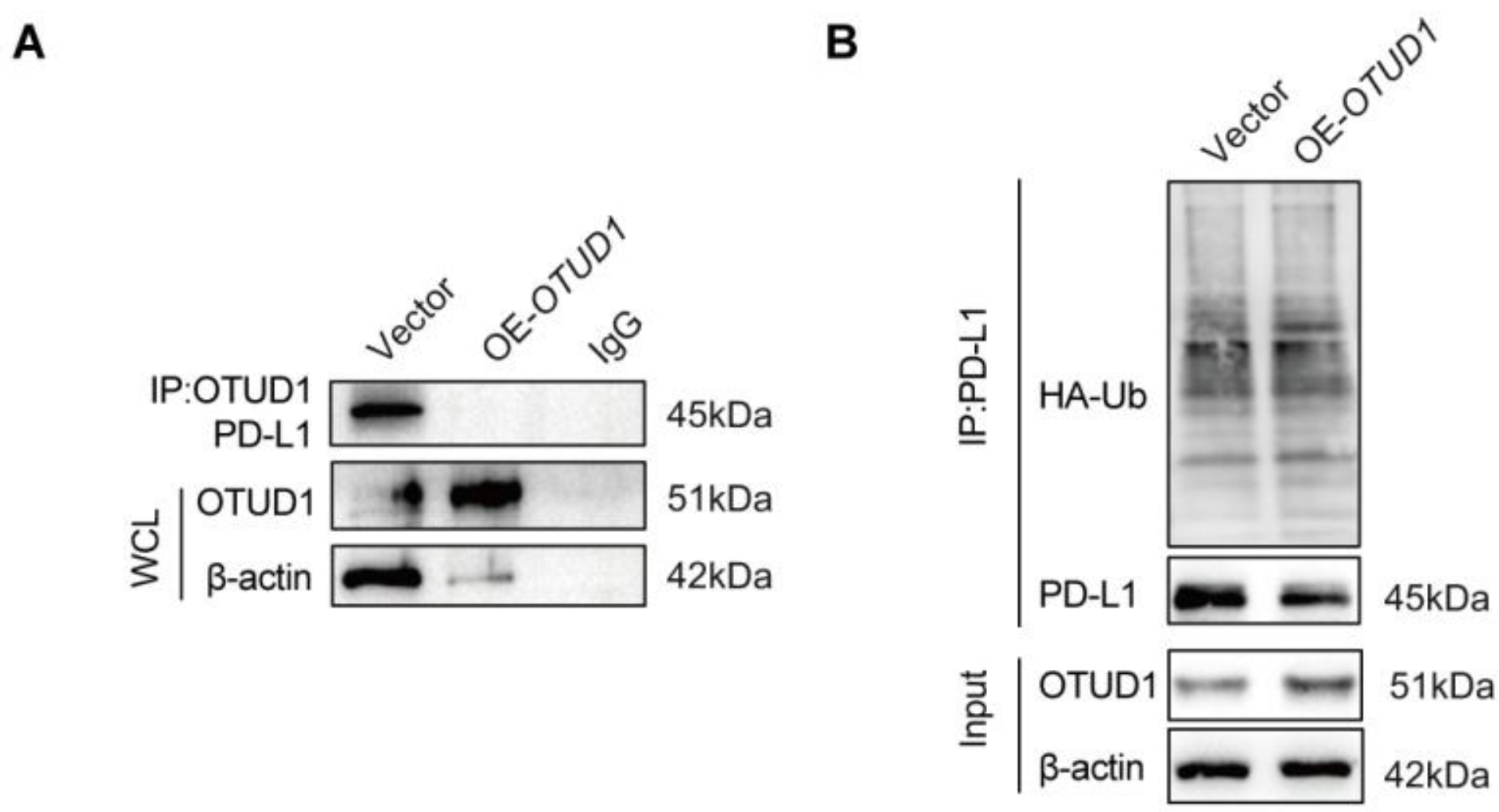

Figure S6 OTUD1 does not bind to PD-L1 to affect its polyubiquitination status. (A) Co-immunoprecipitation (Co-IP) assay was performed to examine the interaction between HA-OTUD1 and FLAG-PD-L1. (B) The effect of OTUD1 overexpression on PD-L1 polyubiquitination. Relative ubiquitination abundance is shown as mean ± SEM from three independent experiments.

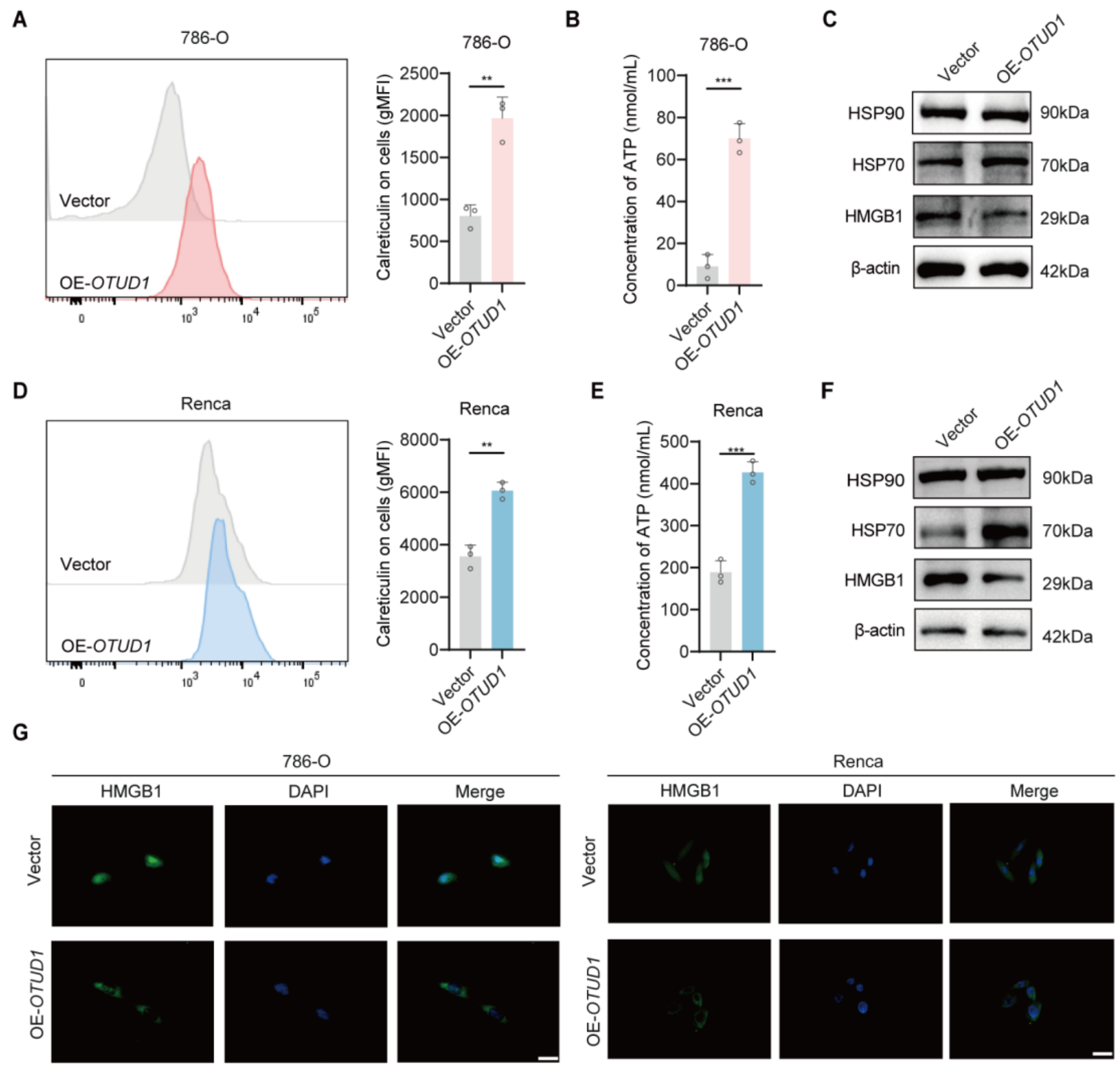

Figure S7 OTUD1 triggers the release of DAMPs and stimulates an immune response *in vivo*. (A-B) Effects of OTUD1 overexpression on surface calreticulin and

(C) extracellular ATP level in the medium of 786-O cells. (D) Immunoblots of the protein expression level of common DAMPs, including HSP90, HSP70, and HMGB1, in 786-O cells with overexpressed OTUD1 and control cells. (E-F) Effects of OTUD1 overexpression on surface calreticulin and (G) extracellular ATP level in the medium of Renca cells. (H) Immunoblots of the protein expression level of common DAMPs, including HSP90, HSP70, and HMGB1, in Renca cells with overexpressed OTUD1 and control cells. (I) Representative fluorescent microscopy images of HMGB1 subcellular localization in 786-O cells and (J) Renca cells with overexpressed OTUD1 expression and control cells. Representative images from two independent experiments are shown. Significance was assessed using a two-tailed paired Student' s *t*-test (A, B, D, E). Data are representative of at least three (C, F) independent experiments with similar results.

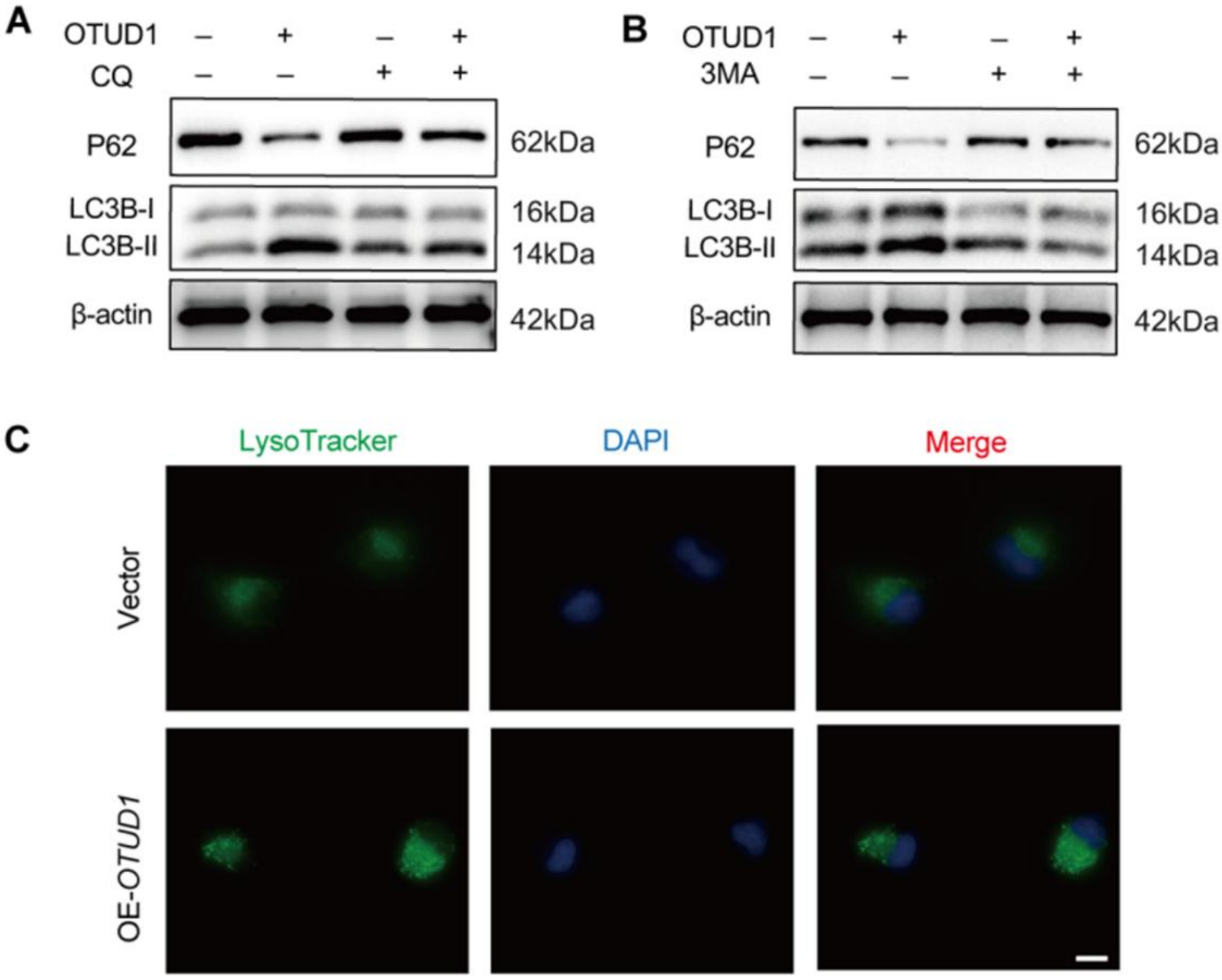

Figure S8 OTUD1 regulates autophagosome formation, lysosomal biogenesis, and OTUD1 activity. (A) CQ and (B) 3MA were used to block the autophagy-lysosomal pathway. Immunoblots of the protein expression level of P62 and LC3B in 786-O cells overexpressing OTUD1 and control cells. (C) LysoTracker in 786-O cells with overexpressed OTUD1 expression and control cells. Representative images from two independent experiments are shown. Scale bar = 20 μm. Data are representative of at least three (A-D) independent experiments with similar results.

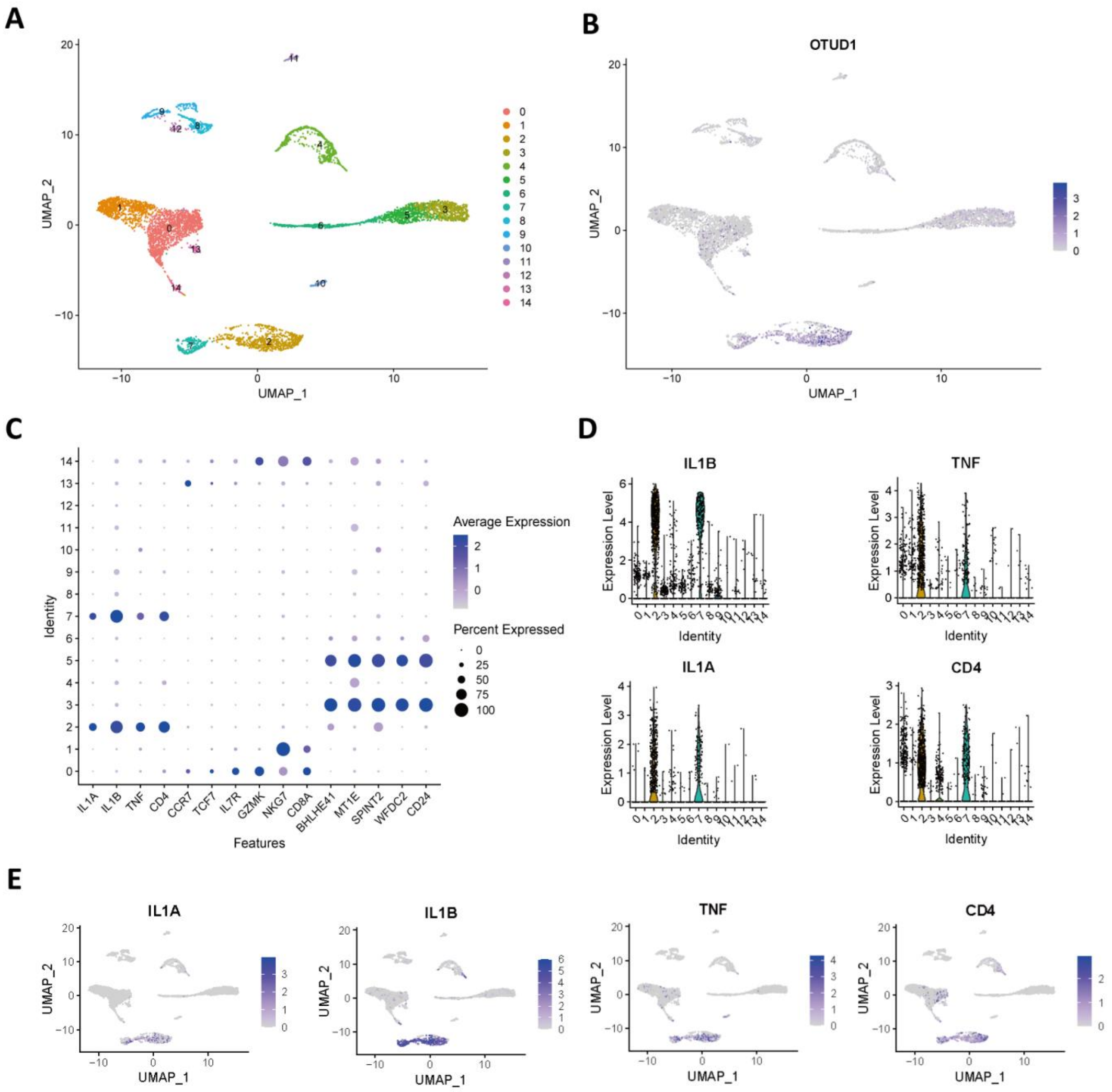

Figure S9 The expression pattern of OTUD1 in lymphocytes of KIRC patients based on the single-cell sequencing analysis. We identified 14 distinct cell clusters and Cluster 6 was identified as Th17 cells based on the expression of *IL1A*, *IL1B*, *TNF*, and *CD4*, consistent with the UMAP annotations. (A) UMAP plot shows the cellular subtypes of KIRC into clusters based on the single-cell sequencing analysis of publicly available data (GEO number: GSE171306). (B) The expression of OTUD1 in different cellular subtypes, with gene expression colored according to the Log2 scale shown on the right of each panel. UMAP_1 and UMAP_2 represent the first two UMAP dimensions. (C) Dot plots showing average expression of known markers for various immune cells in all clusters, including Th17 cells (*IL1A, IL1B, TNF, CD4*), T cells (*CCR7, TCF7, IL7R, GZMK, NKG7, CD8A*), and B cells (*BHLHE41, MT1E, SPINT2, WFDC2, CD24*). The dot size represents percent of cells expressing the genes in each cluster. The expression intensity of markers is shown. (D) OTUD1 influences the tumor-infiltrating Th17 cells in CRC patients by single-cell and spatial analysis. The normalized expression levels of Th17 cell markers in all clusters showed by violin plot and (E) UMAP dimension reduction plots, with gene expression colored according to the Log2 scale shown on the right of each panel. UMAP_1 and UMAP_2 represent the first two UMAP dimensions.

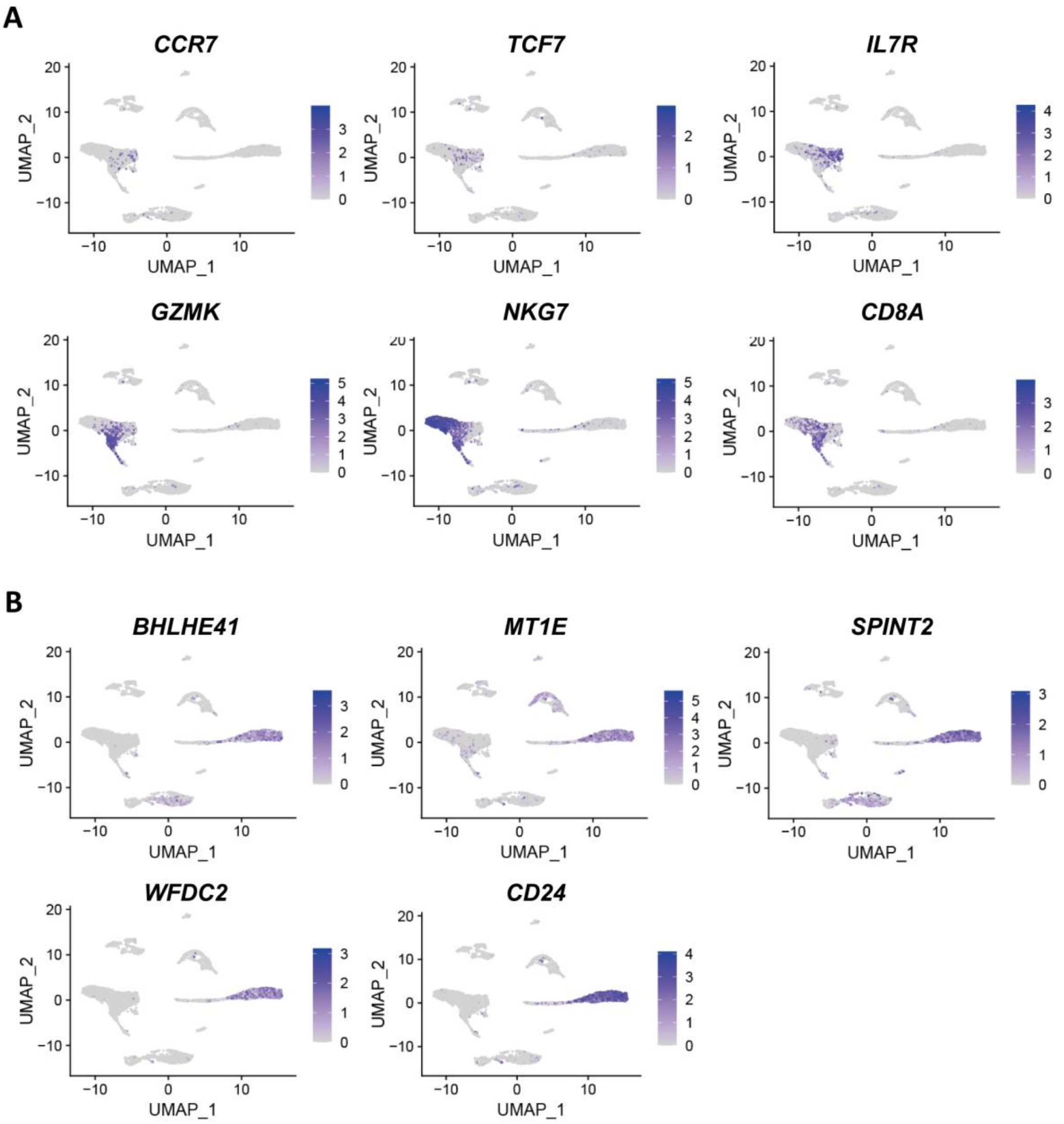

Figure S10 OTUD1 influences the tumor-infiltrating CD8$^+$ T cells and B cells in KIRC patients by single-cell and spatial analysis. UMAP plot shows the cellular subtypes of KIRC into clusters based on the single-cell sequencing analysis of publicly available data (GEO number: GSE171306). OTUD1 was highly enriched in clusters 2 and 7, which were annotated as CD8$^+$ T cells (Part IV, markers: *CCR7*, *TCF7*, *IL7R*, *GZMK*, *NKG7*, *CD8A*) and B cells (Part V, markers: *BHLHE41*, *MT1E*, *SPINT2*, *WFDC2*, *CD24*). (A) UMAP dimension reduction plots showed the normalized expression levels of *CCR7*, *TCF7*, *IL7R*, *GZMK*, *NKG7*, *CD8A* for CD8$^+$ T cells; and (B) *BHLHE40*, *MT1E*, *SPINT2*, *WFDC2*, *CD24* for B cells, Gene expression was colored according to the Log_2 scale shown on the right of each panel. UMAP_1 and UMAP_2 represent the first two UMAP dimensions.

FIG 4A

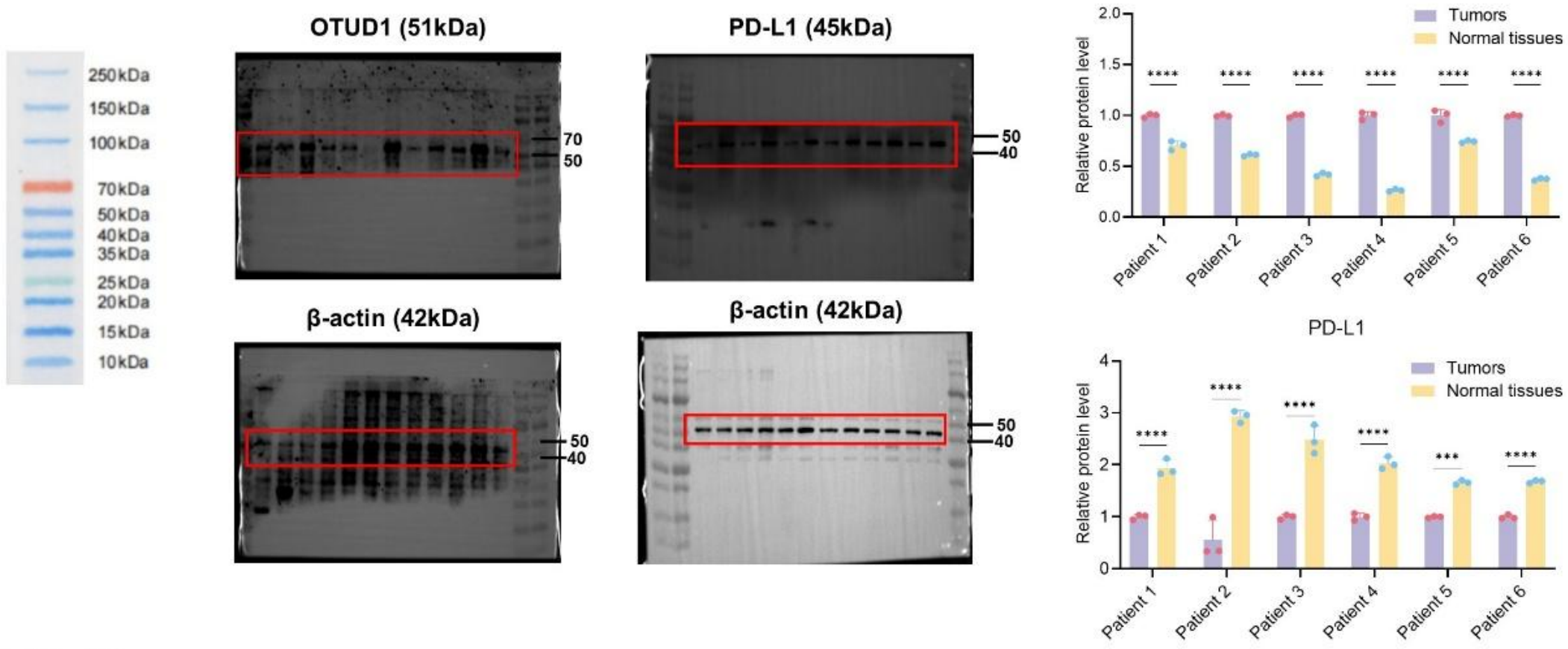

FIG 4C

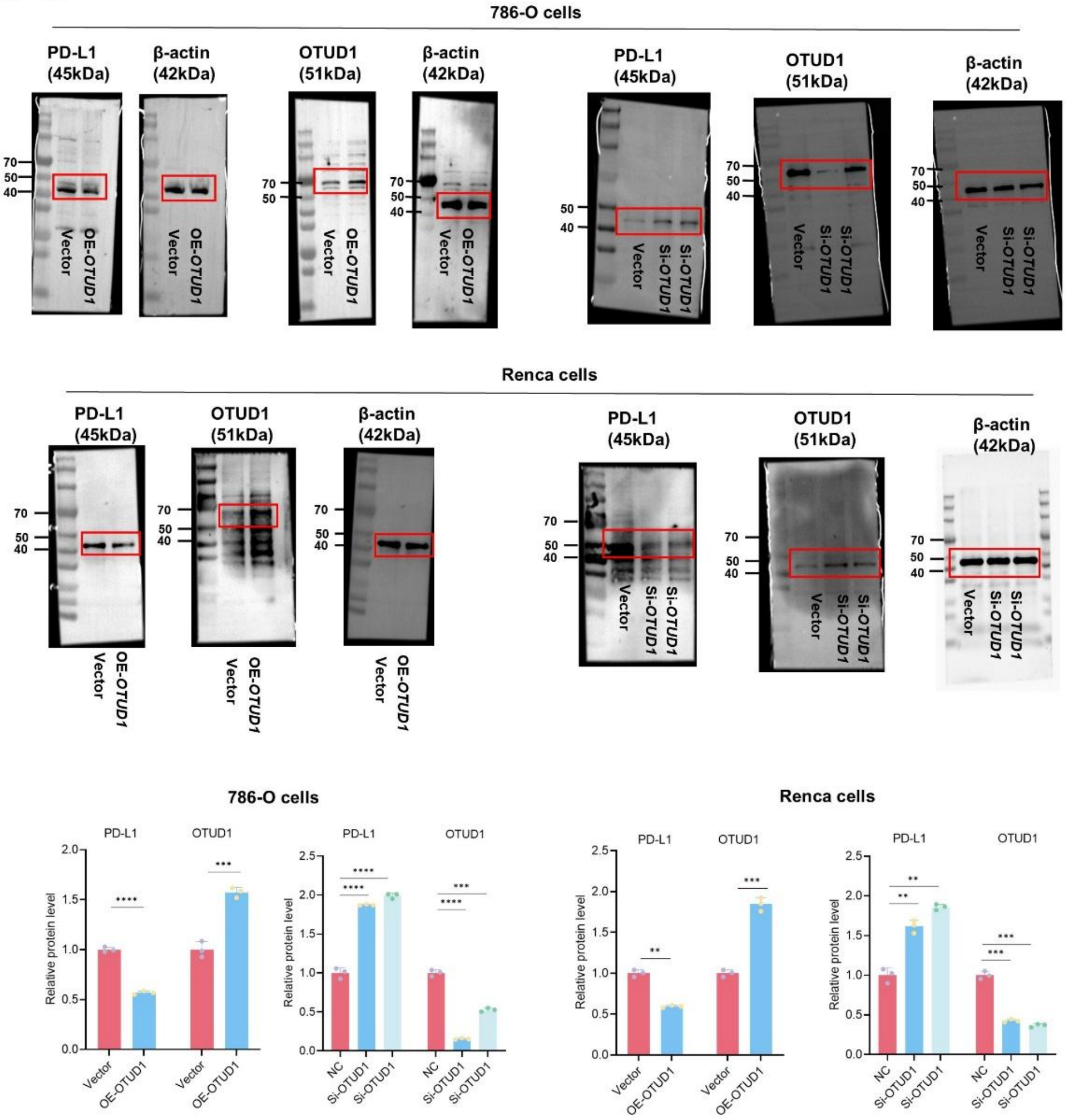

Supplementary Figure S11 Full scan (uncropped) western blots from main Figure 4 Uncropped full scans of western blots from the corresponding cropped western blots shown within the main text. Molecular weight markers are indicated. Figure subpanel indicated for each respective blot.

FIG 6A

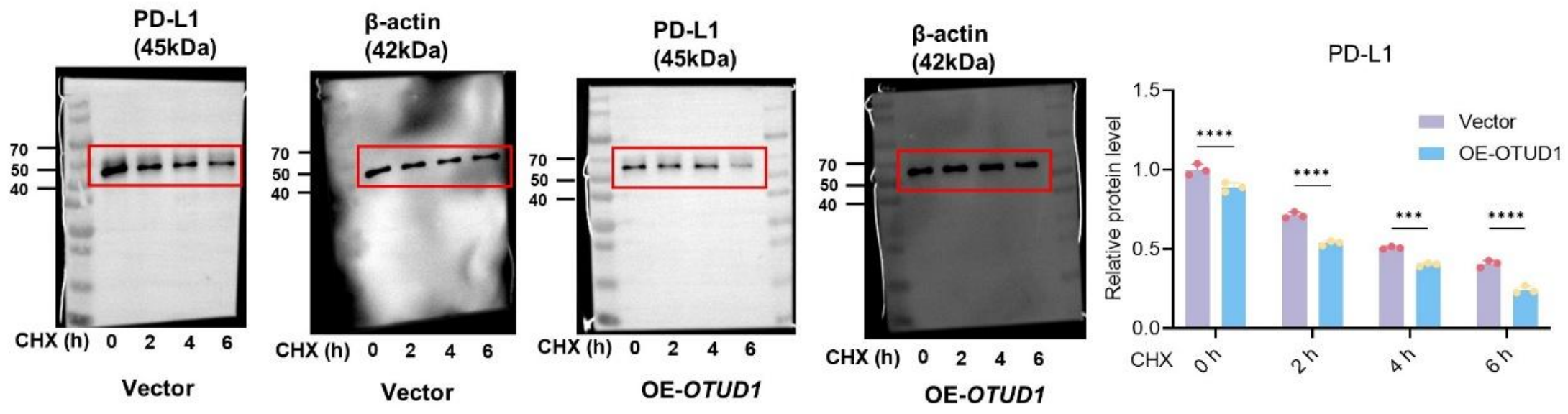

FIG 6C

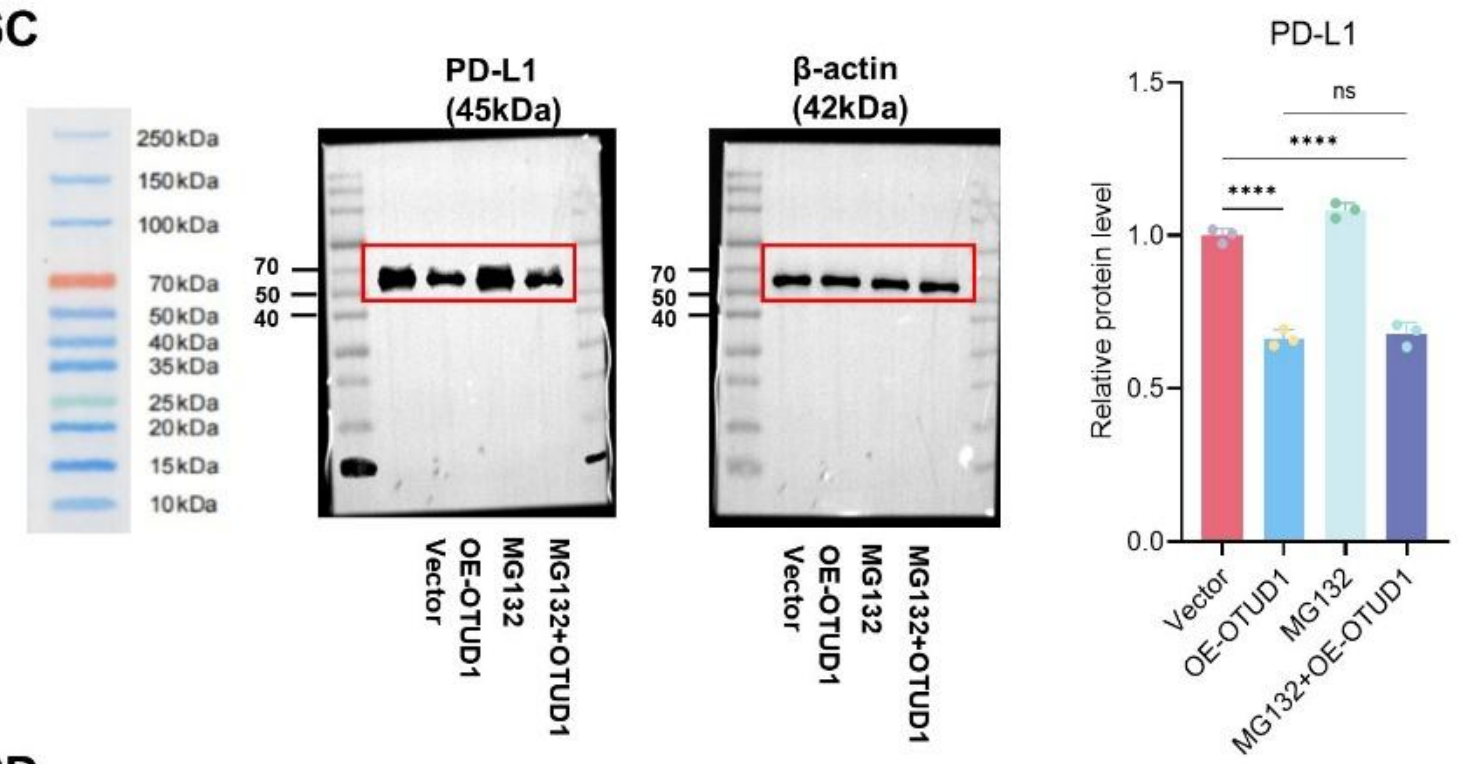

FIG 6D

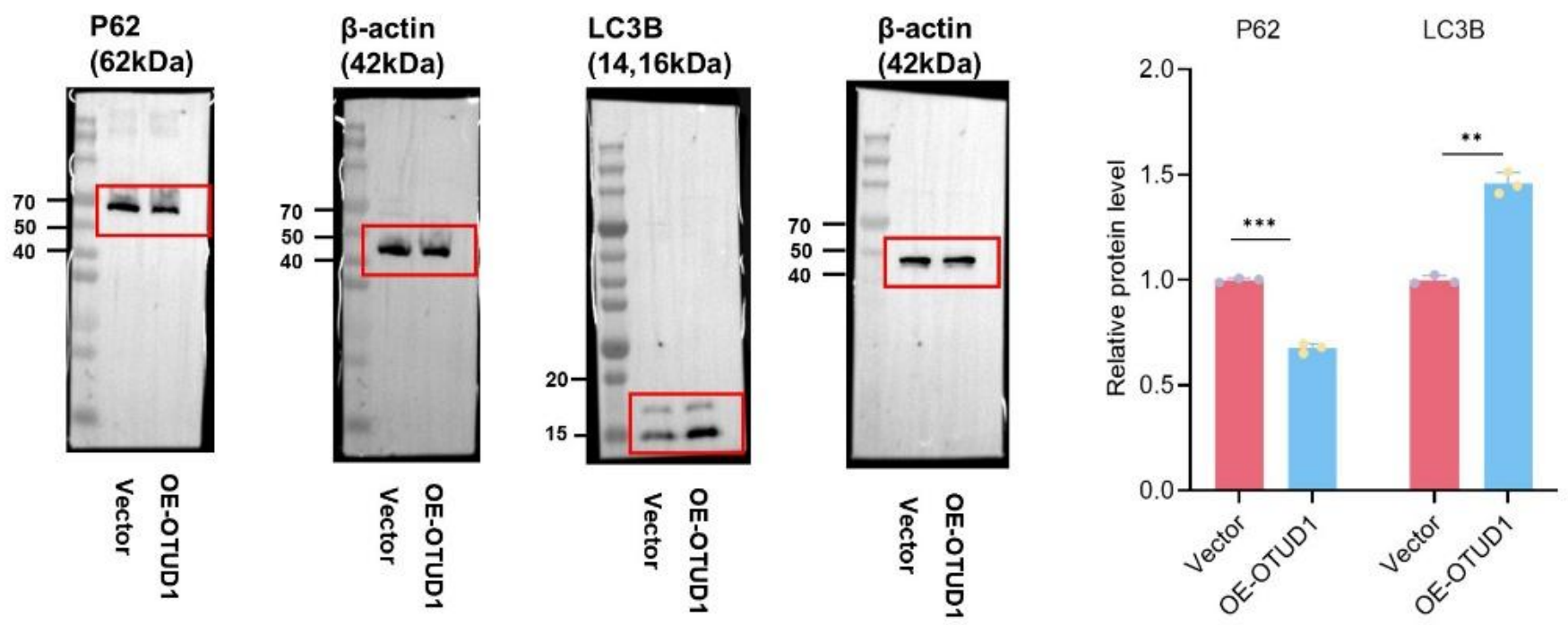

FIG 6H

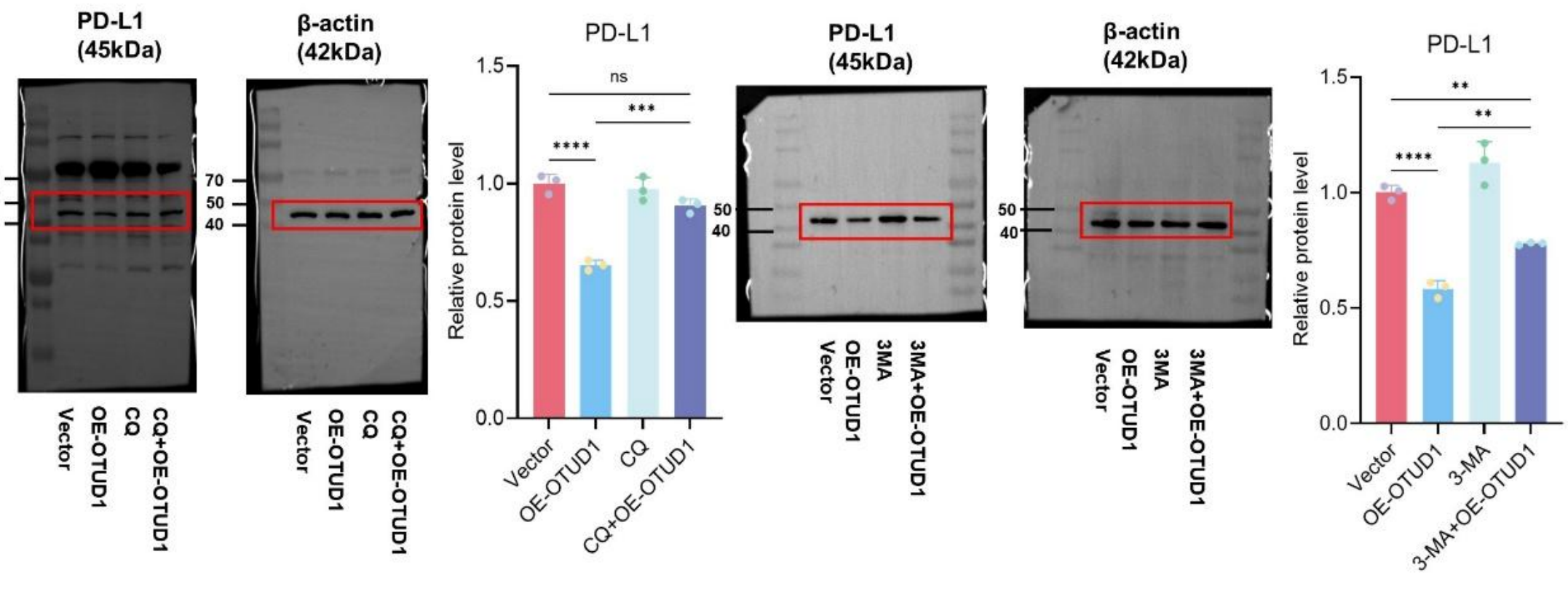

Supplementary Figure S12 Full scan (uncropped) western blots from main Figure 6 Uncropped full scans of western blots from the corresponding cropped western blots shown within the main text. Molecular weight markers are indicated. Figure subpanel indicated for each respective blot.

FIG 7B

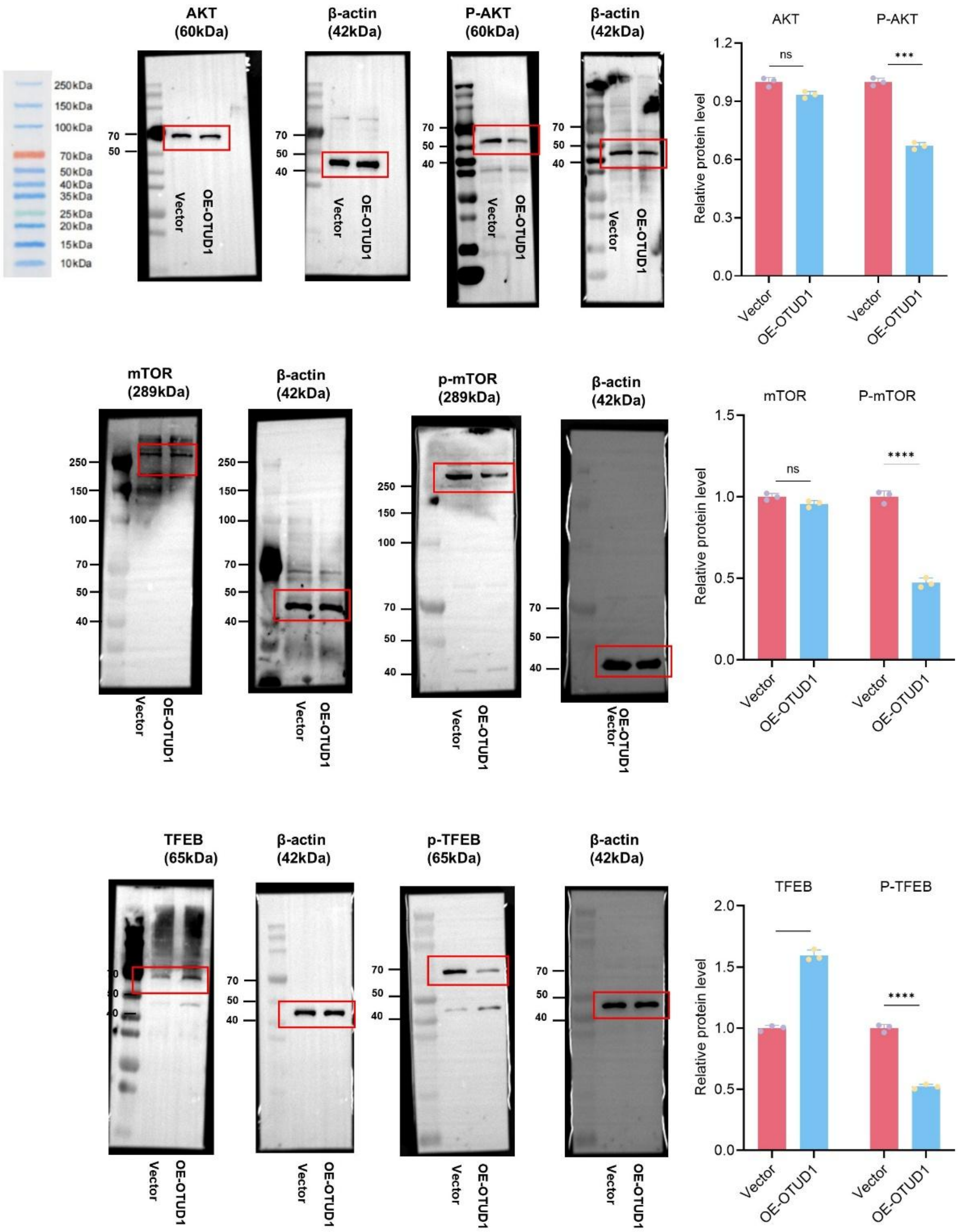

Supplementary Figure S13 Full scan (uncropped) western blots from main Figure 7B Uncropped full scans of western blots from the corresponding cropped western blots shown within the main text. Molecular weight markers are indicated. Figure subpanel indicated for each respective blot.

FIG 7C

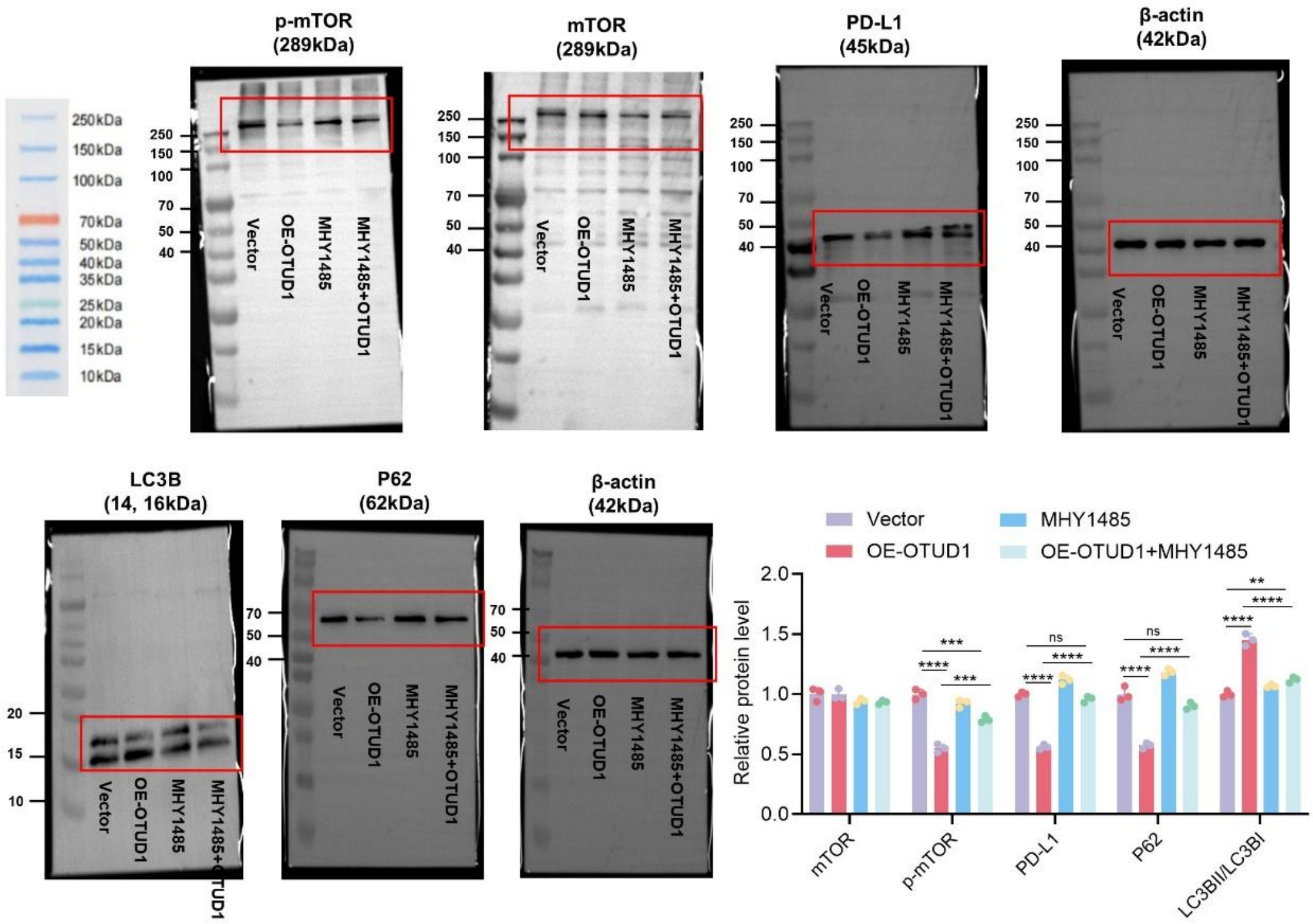

FIG 7D

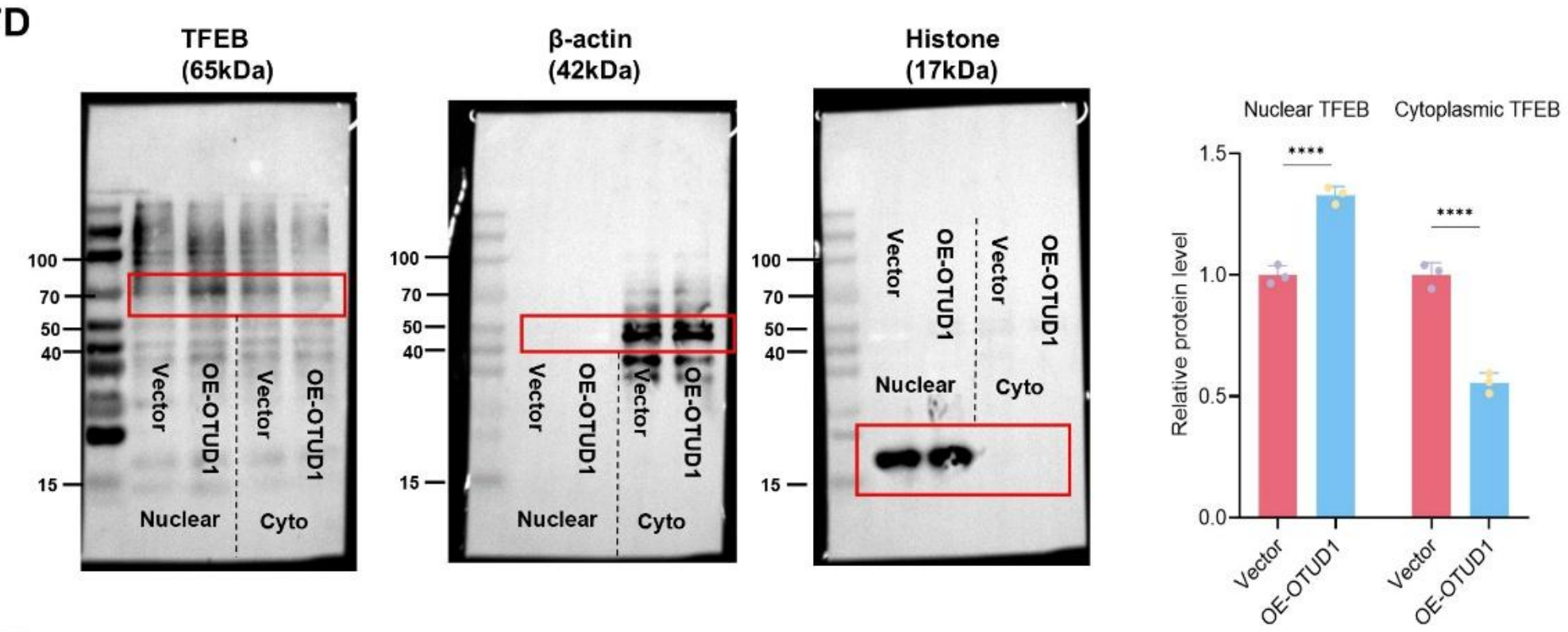

FIG 7H

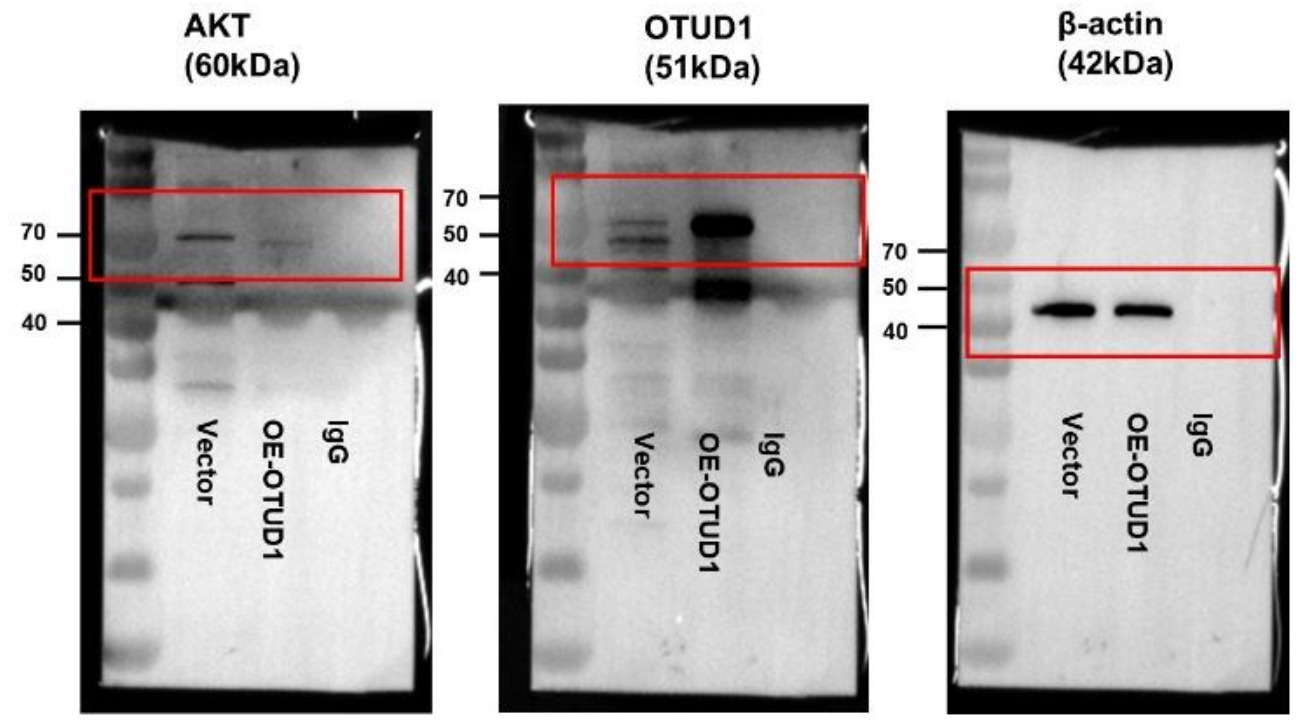

Supplementary Figure S14 Full scan (uncropped) western blots from main Figure 7C-H Uncropped full scans of western blots from the corresponding cropped western blots shown within the main text. Molecular weight markers are indicated. Figure subpanel indicated for each respective blot.

Figure S3B

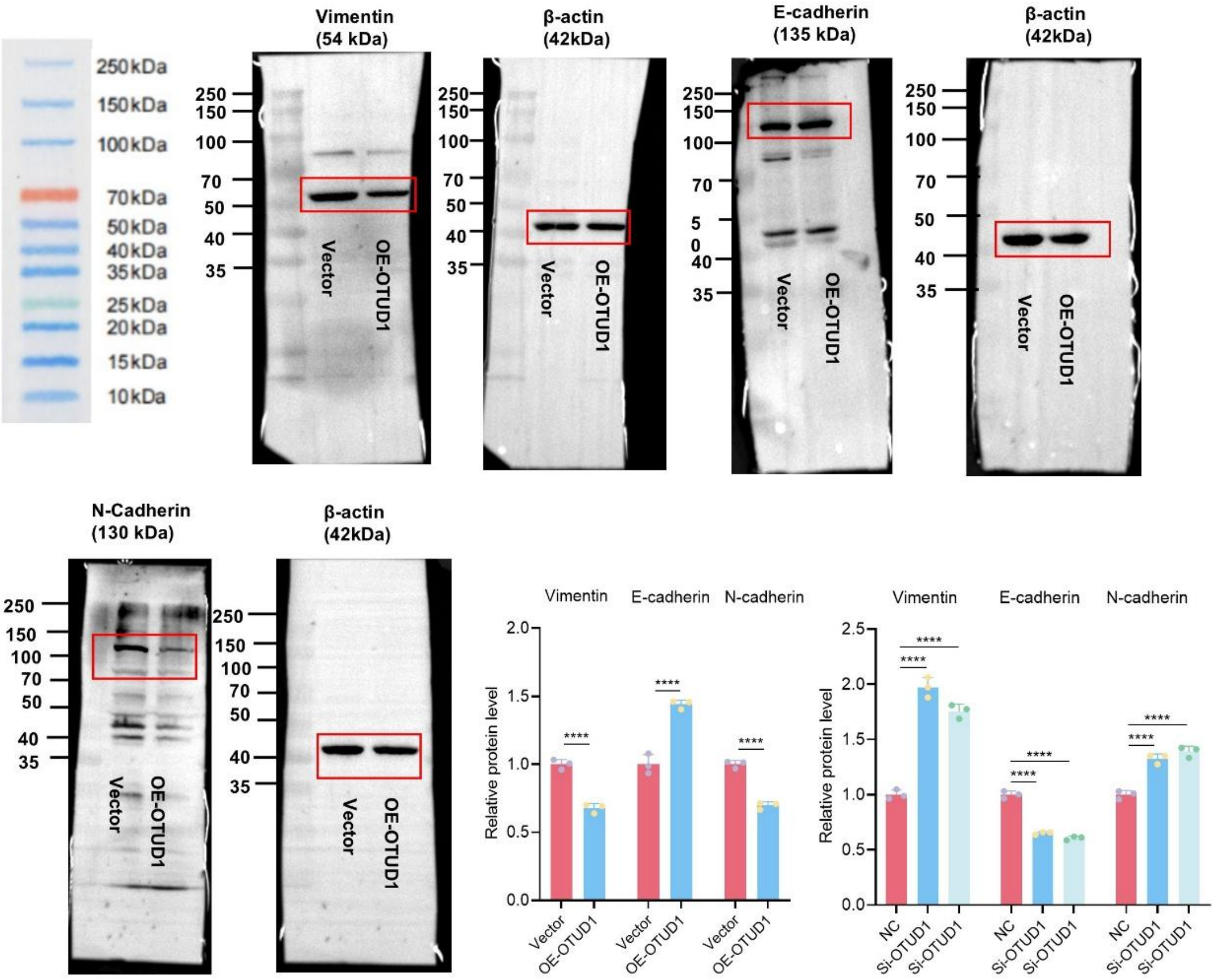

Figure S3D

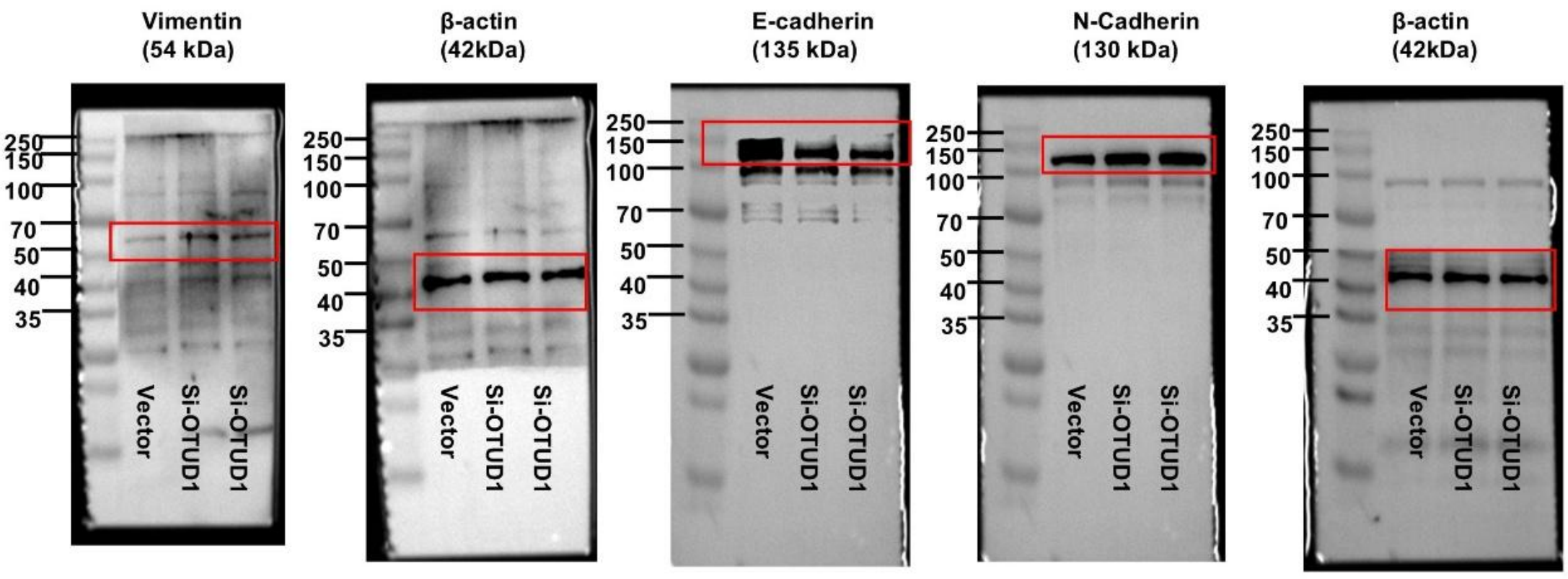

Supplementary Figure S15 Full scan (uncropped) western blots from Figure S3 Uncropped full scans of western blots from the corresponding cropped western blots shown within the main text. Molecular weight markers are indicated. Figure subpanel indicated for each respective blot.

Figure S6A

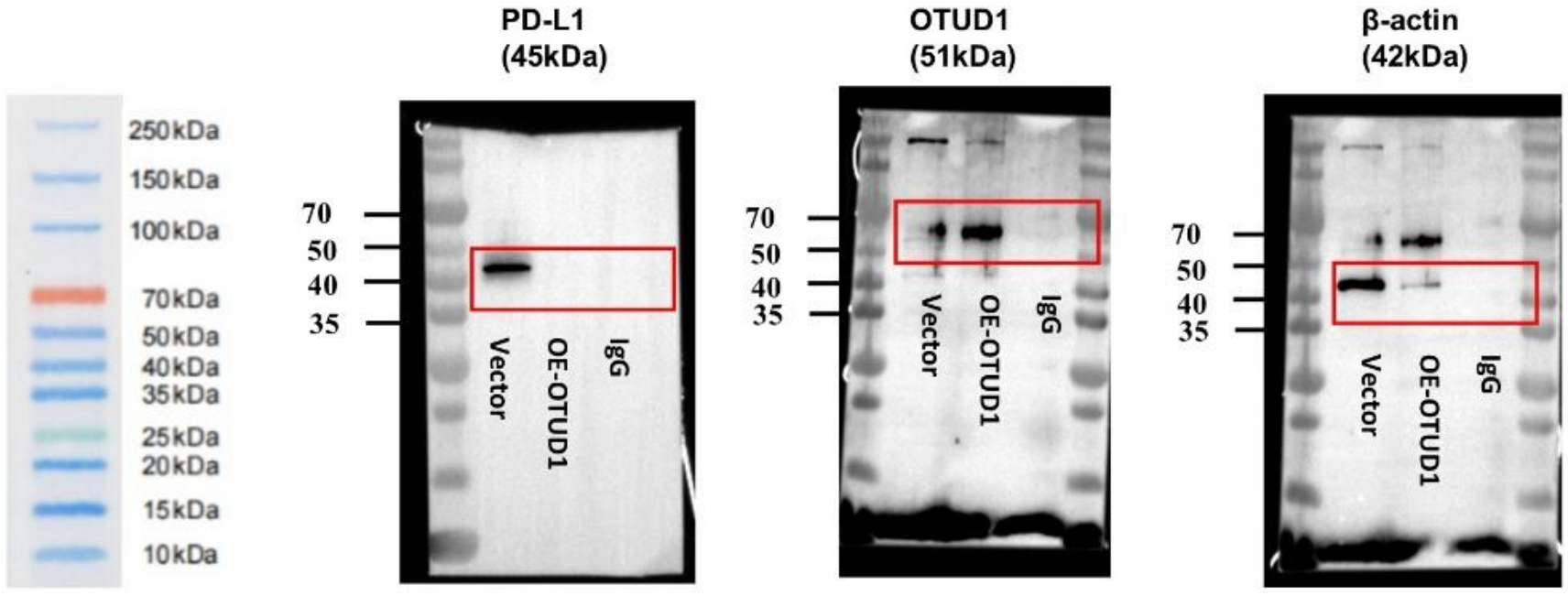

Supplementary Figure S16 Full scan (uncropped) western blots from Figure S6A
Uncropped full scans of western blots from the corresponding cropped western blots shown within the main text. Molecular weight markers are indicated. Figure subpanel indicated for each respective blot.

FIG S7C

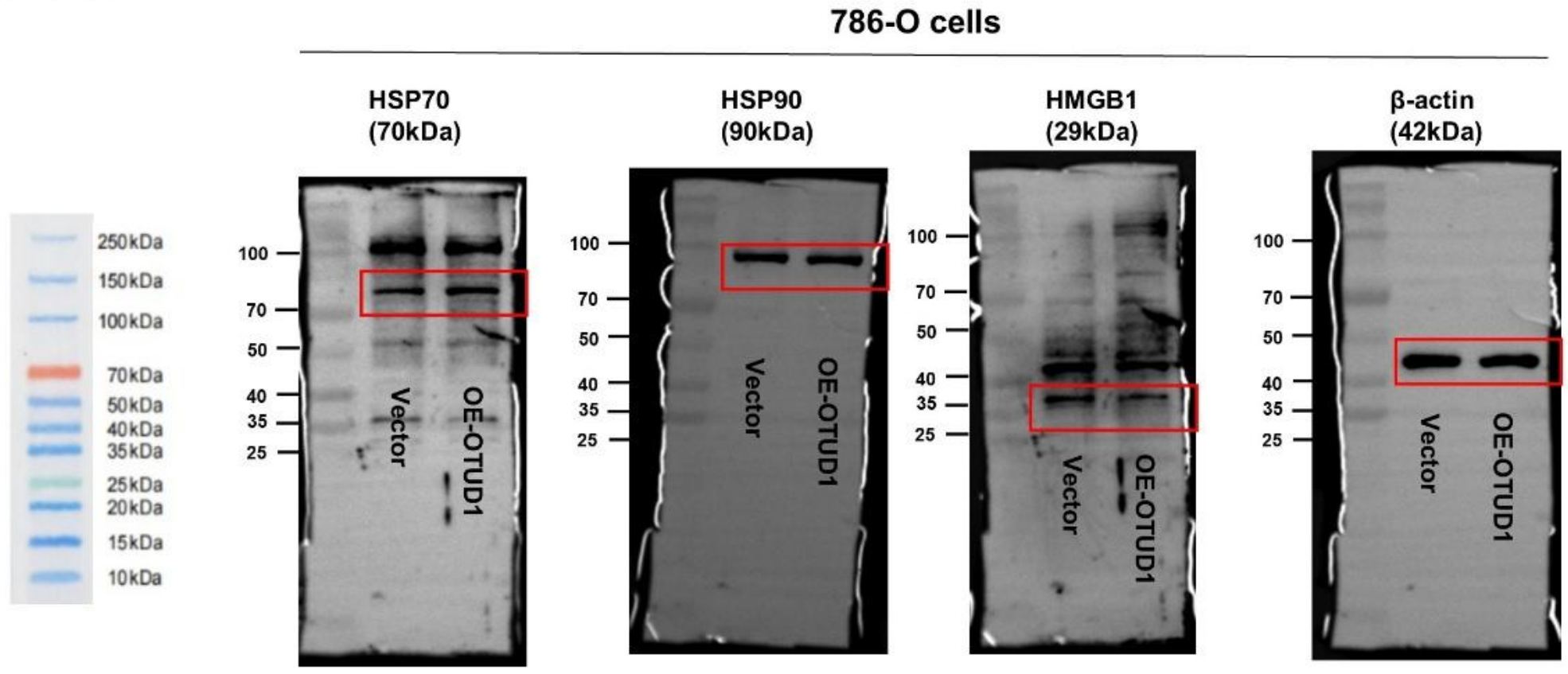

FIG S7F

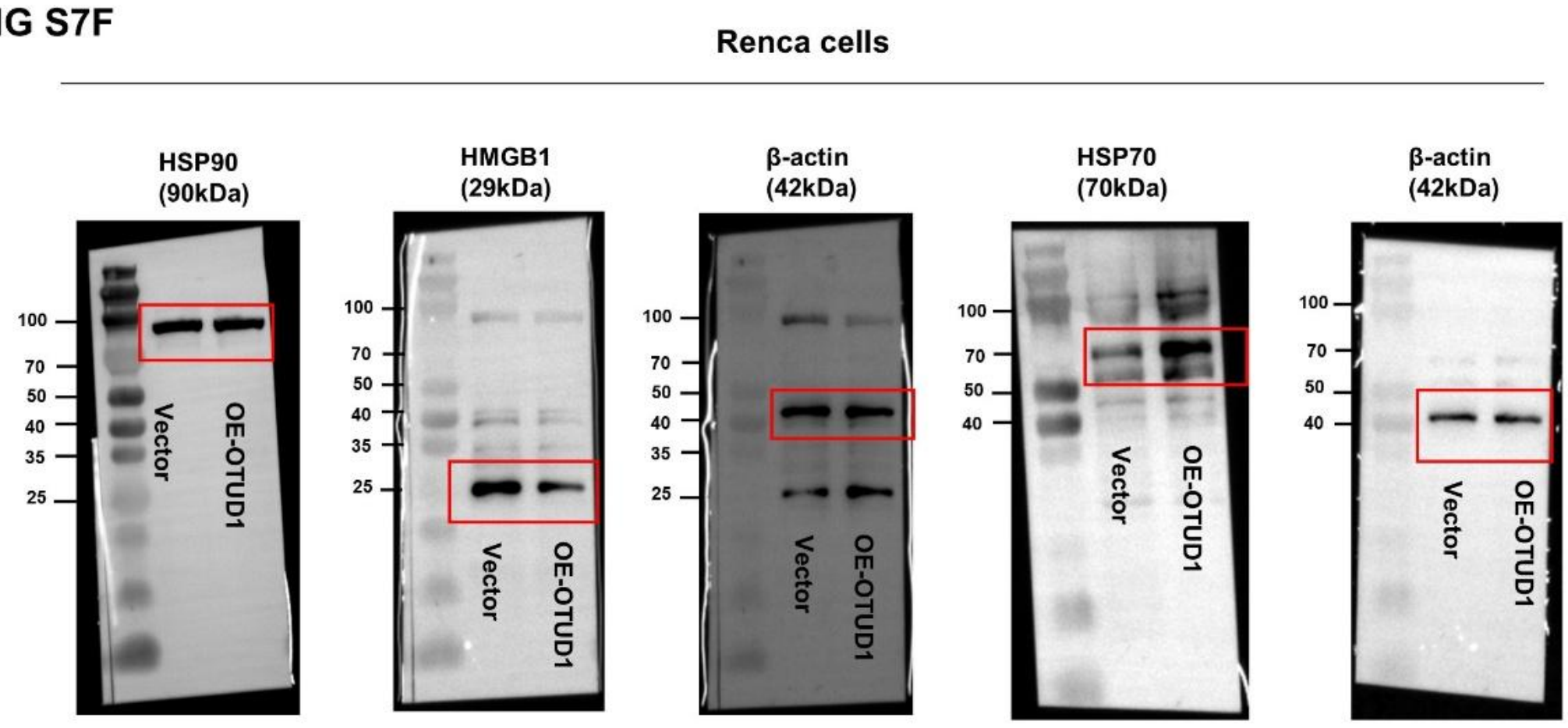

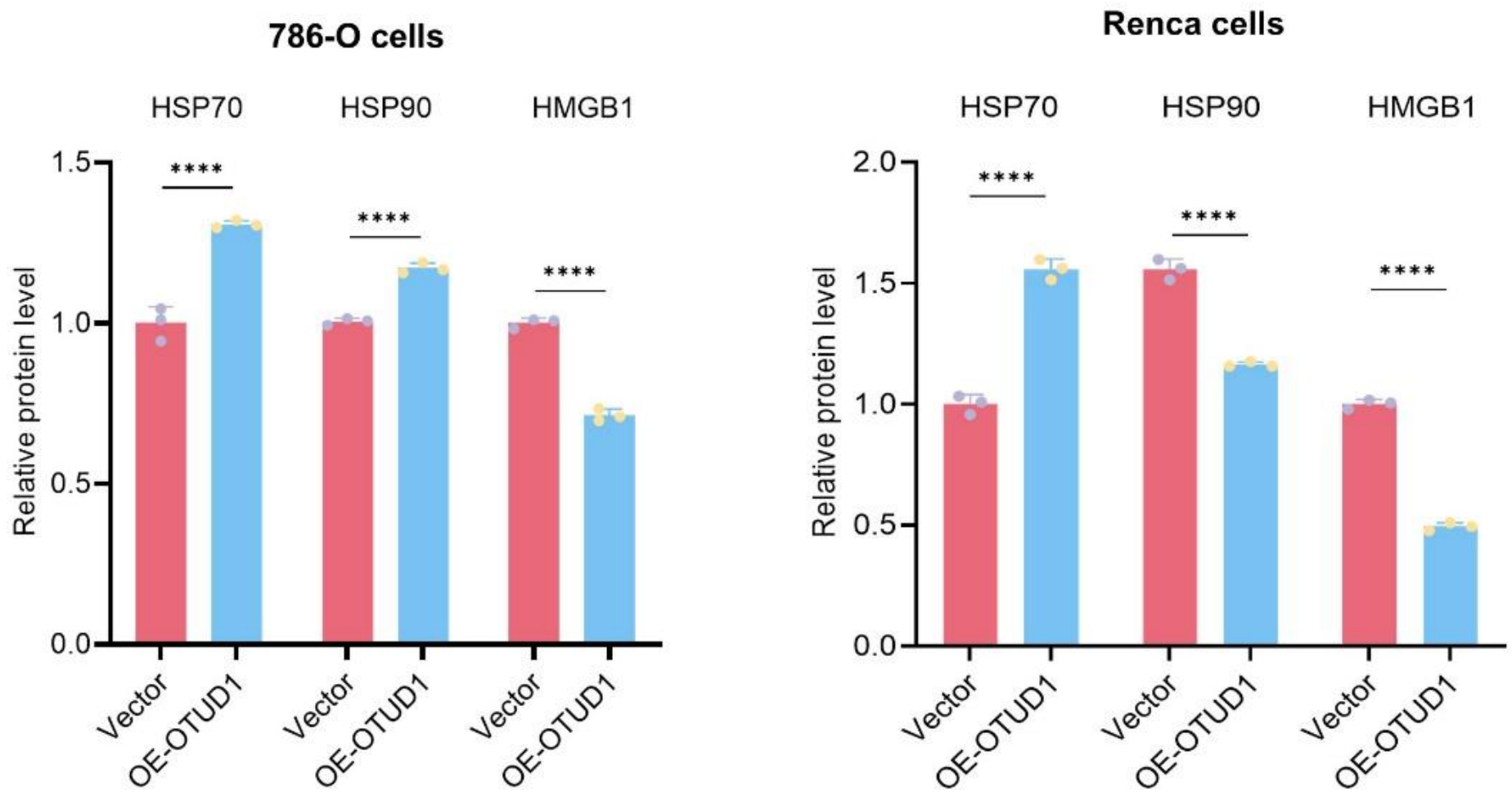

Supplementary Figure S17 Full scan (uncropped) western blots from Figure S7
Uncropped full scans of western blots from the corresponding cropped western blots shown within the main text. Molecular weight markers are indicated. Figure subpanel indicated for each respective blot.

Figure S8A

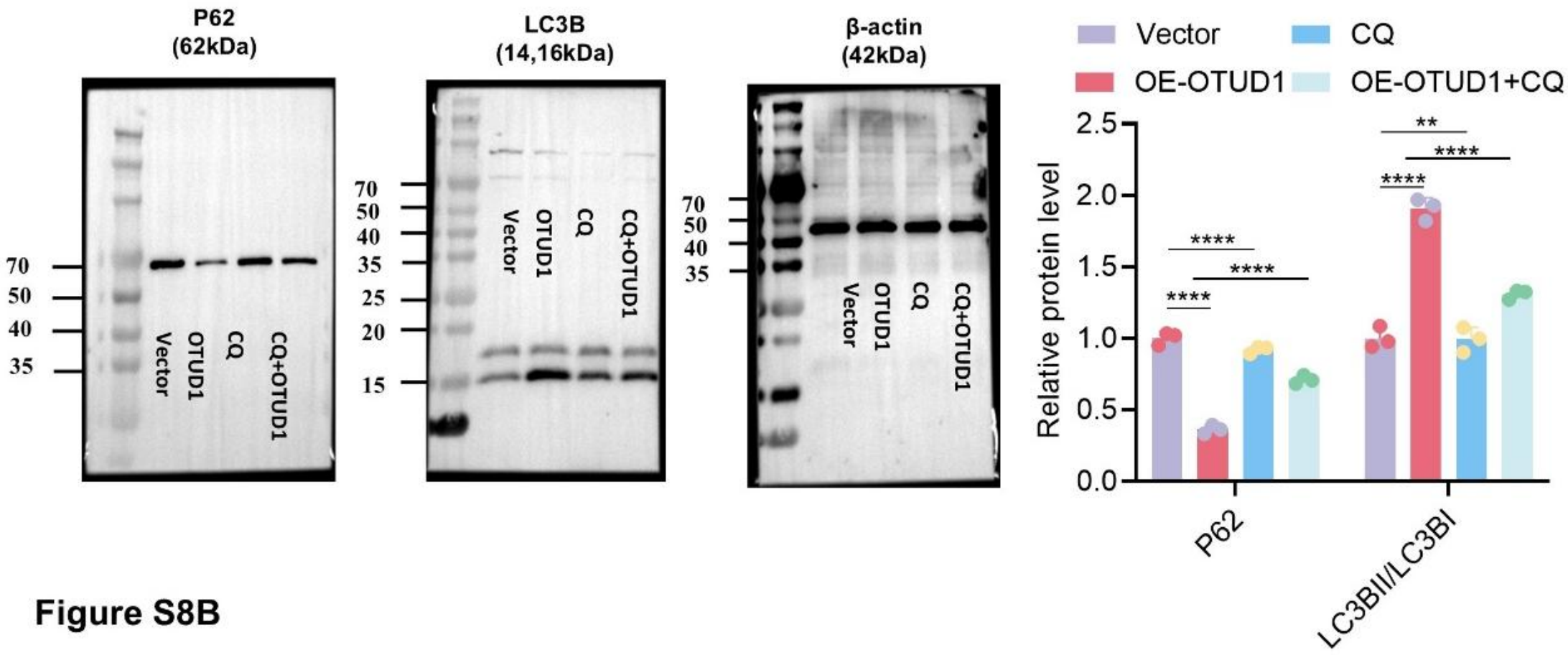

Figure S8B

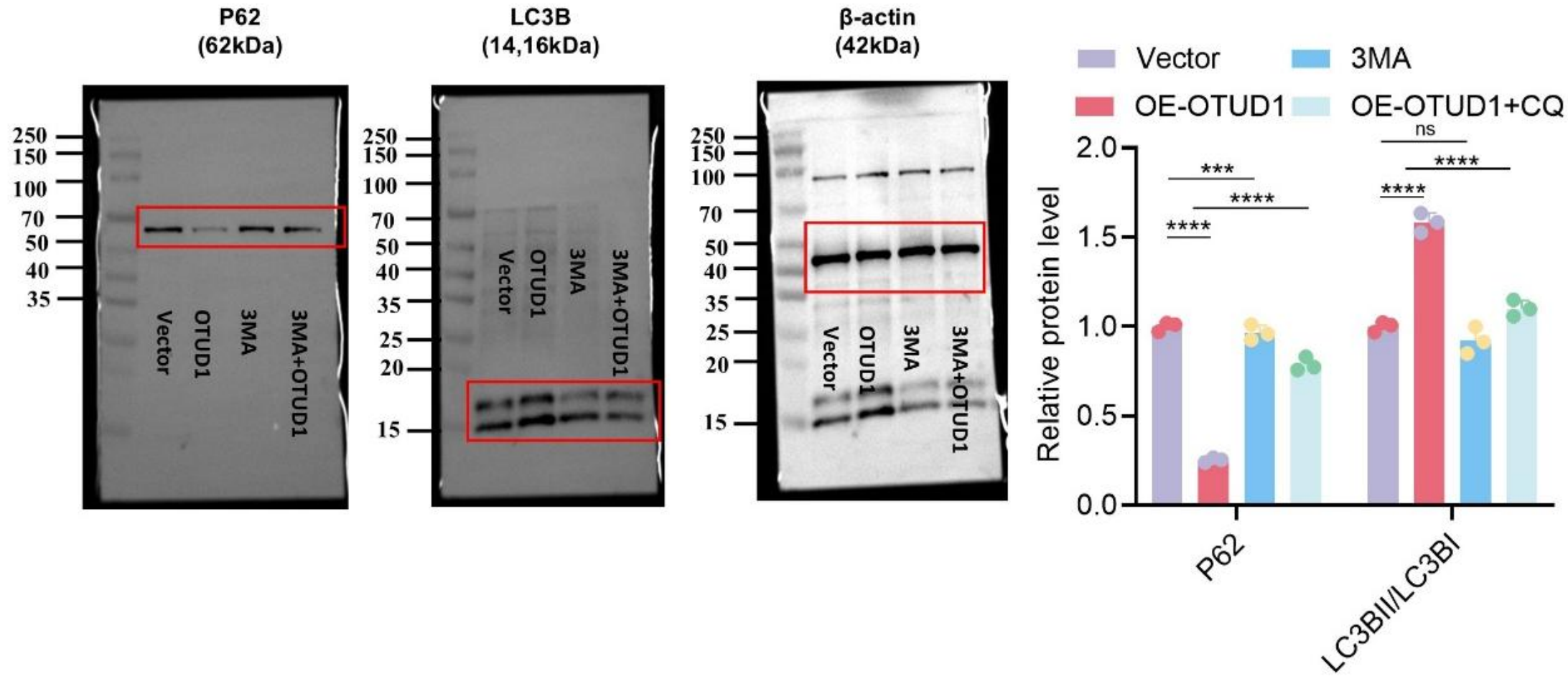

Supplementary Figure S18 Full scan (uncropped) western blots from Figure S8
Uncropped full scans of western blots from the corresponding cropped western blots shown within the main text. Molecular weight markers are indicated. Figure subpanel indicated for each respective blot.

Supplementary Table S1. Antibodies used for western blotting.

Antibody	Company	Catalog number	Dilution
OTUD1 Polyclonal antibody	Proteintech	29921-1-AP	1:1000
PD L1 Ab	Abways	CY5980	1:1000
E-cadherin	Wanleibio	WL01482	1:1000
N-cadherin	Wanleibio	WL01047	1:1000
Vimentin	Proteintech	10366-1-AP	1:2000
HSP70	Wanleibio	WL010	1:500
HSP90	Wanleibio	WL01763	1:1000
HMGB1	Wanleibio	WL03023	1:500
Phospho-mTOR (Ser2448) Rabbit pAb	Zenbio	#381557	1:1000
mTOR Rabbit mAb	Zenbio	#R380411	1:1000
Akt Polyclonal Antibody	Baijia	IPB0950	1:500
p-AKT(Ser473)	Wanleibio	WLP001a	1:1000
p-AKT(T308)	Wanleibio	WLA0352	1:500
Phospho-TFEB (Ser211) Antibody	Abmart	TA3708	1:1000
Histone H1.2 Polyclonal antibody	Proteintech	19649-1-AP	1:2000
TFEB Rabbit mAb	Zenbio	#R27339	1:500
LC3B	Nature Biosciences	A27936	1:1000
SQSTM1/p62 Rabbit pAb	Zenbio	#380612	1:1000
Recombinant Anti-beta Actin antibody (HRP Conjugated)	Servicebio	P60710	1:3000

Supplementary Table S2. Primers used for RT-qPCR.

Gene	Sequence (5' to 3')	
SNAI1e	Forward	GTGTGTTCTGGTCTGTTCTAGAACC
	Reverse	GGAAGACTGCCTGGCACACA
H19	Forward	GAAACAGACCCGCTTCTTGC
	Reverse	ATGACATGGTCCGGTGTGAC
NEAT1	Forward	ACAGCATTCCTGTCTGCGAA
	Reverse	GACTTCAGGCTCCAGCCATT
SMAD4	Forward	CCACCAAAACGGCCATCTT
	Reverse	TGGAAATGGGAGGCTGGAA
PAI-1	Forward	CACAAATCAGACGGCAGCACT
	Reverse	CATCGGGCGTGGTGAACTC
CTGF	Forward	TTGCGAAGCTGACCTGGAAGAGAA
	Reverse	AGCTCGGTATGTCTTCATGCTGGT
SMAD7	Forward	TCCAGATGCTGTGCCCTTCC
	Reverse	GTCCGAATTGAGCTGTCCG
TBRI	Forward	ACGGCGTTACAGTGTTTCTG
	Reverse	GCACATACAAACGGCCTATCT
SLC9A8	Forward	TTTCTGGGTCAGGCTGATGT
	Reverse	CCACTGGATCGACAGCAGAT
RNF144	Forward	TCCCACGTGGCTACTTGTTC
	Reverse	CGGTTTGGAACATTCCTTGGC
UBE2V1	Forward	CAAGCAAGAGCGACGCAAG
	Reverse	CCCAGCTAACTGTGCCATCT
PEDS1	Forward	GGAGCTGCCCATTGCTTTCA
	Reverse	CCCGTTGGTCTCGATGAAGT
CEBPB	Forward	GCTTAAAGATGTTCCTACGGGC
	Reverse	CCCCCAAAAGGCTTTGTAACC
SCREEM1	Forward	TGTTGCTCAGGCTGGTCTTGAA
	Reverse	TCAGCTGAGCACAATGGTTCACA
TRERNA1	Forward	GGGTCTCACTCTCTCAAATCGG
	Reverse	CAGAGACGGAGAAGATGAACAGGG
SNAI1	Forward	ACCACTATGCCGCGCTCTT
	Reverse	GGTCGTAGGGCTGCTGGAA
GAPDH	Forward	TGCACCACCAACTGCTTAGC
	Reverse	GGCATGGACTGTGGTCATGAG
	Reverse	CATAGTGGTCGAGGCACTGG
